# A comprehensive human embryo reference tool using single-cell RNA-sequencing data

**DOI:** 10.1038/s41592-024-02493-2

**Published:** 2024-11-14

**Authors:** Cheng Zhao, Alvaro Plaza Reyes, John Paul Schell, Jere Weltner, Nicolás M. Ortega, Yi Zheng, Åsa K. Björklund, Laura Baqué-Vidal, Joonas Sokka, Ras Trokovic, Brian Cox, Janet Rossant, Jianping Fu, Sophie Petropoulos, Fredrik Lanner

**Affiliations:** 1 Department of Clinical Science, Intervention and Technology, Karolinska Institutet, and Division of Obstetrics and Gynecology, Karolinska Universitetssjukhuset, Stockholm, Sweden; 2 Department of Integrative Pathophysiology and Therapy, Andalusian Molecular Biology and Regenerative Medicine Centre (CABIMER), Seville, Spain; 3 Stem Cells and Metabolism Research Program, University of Helsinki, Helsinki, Finland; 4 Folkhälsan Research Center, Helsinki, Finland; 5 Department of Mechanical Engineering, University of Michigan, Ann Arbor, MI USA; 6 Department of Biomedical and Chemical Engineering, Syracuse University, Syracuse, NY USA; 7 Department of Cell and Molecular Biology, National Bioinformatics Infrastructure Sweden, Science for Life Laboratory, Uppsala University, Uppsala, Sweden; 8 Department of Physiology, Faculty of Medicine, University of Toronto, Toronto, Ontario Canada; 9 Program in Developmental and Stem Cell Biology, Hospital for Sick Children, Toronto, Ontario Canada; 10 Department of Cell and Developmental Biology, University of Michigan Medical School, Ann Arbor, MI USA; 11 Department of Biomedical Engineering, University of Michigan, Ann Arbor, MI USA; 12 Département de Médecine, Université de Montréal, Montreal, Quebec Canada; 13 Centre de Recherche du Centre Hospitalier de l’Université de Montréal, Axe Immunopathologie, Montreal, Quebec Canada; 14 Ming Wai Lau Center for Reparative Medicine, Stockholm Node, Karolinska Institutet, Stockholm, Sweden

**Keywords:** Embryogenesis, Classification and taxonomy, Embryonic stem cells

## Abstract

Stem cell-based embryo models offer unprecedented experimental tools for studying early human development. The usefulness of embryo models hinges on their molecular, cellular and structural fidelities to their in vivo counterparts. To authenticate human embryo models, single-cell RNA sequencing has been utilized for unbiased transcriptional profiling. However, an organized and integrated human single-cell RNA-sequencing dataset, serving as a universal reference for benchmarking human embryo models, remains unavailable. Here we developed such a reference through the integration of six published human datasets covering development from the zygote to the gastrula. Lineage annotations are contrasted and validated with available human and nonhuman primate datasets. Using stabilized Uniform Manifold Approximation and Projection, we constructed an early embryogenesis prediction tool, where query datasets can be projected on the reference and annotated with predicted cell identities. Using this reference tool, we examined published human embryo models, highlighting the risk of misannotation when relevant references are not utilized for benchmarking and authentication.

## Abstract (web summary)

This resource integrates different human embryo datasets to create a transcriptional reference map of human embryonic development from zygote to gastrula.

## Main

Studies of early human development are of fundamental importance to promote understanding of how we are built and how human life begins. Such studies can also shed light on reasons of infertility, early miscarriages and congenital disease. Early human embryos have become more accessible as a result of increased reproductive treatments including in vitro fertilization and preimplantation genetic diagnosis^1^. However, studies of human development are still limited by the scarcity of available human embryos that are donated for research and technical and ethical/legal challenges, such as the 14 day rule, associated with studies of human embryos^2^. For these reasons, the use of stem cell-based embryo models mimicking different aspects of human embryogenesis from the zygote stage to gastrulation has the transformative potential for advancing understanding of early human development^2,3^. However, to establish the usefulness of these models, it is critical to validate and benchmark them against human embryos of corresponding developmental stages to ensure their resemblance and fidelity to the in vivo human embryos they aim to model. Such comparison and validation should be conducted at molecular, cellular, morphological and, when possible, functional levels^4,5^. Molecular characterizations of human embryo models are commonly conducted by examining expression levels of individual lineage markers. However, it is increasingly recognized that cell types and their states are not always distinguishable with individual or a limited number of lineage markers, as many cell lineages that codevelop in early human development share the same molecular markers. As such, global gene expression profiling becomes necessary and offers an opportunity for unbiased transcriptome comparison between human embryos models and their in vitro counterparts. Although still limited, there are a few human embryo transcriptome datasets that have been reported during the last 10 years, covering human developmental stages from the fertilization to the gastrulation^6–11^. Efforts have been made to integrate these datasets; however, a well-organized and comprehensive human single-cell RNA-sequencing (scRNA-seq) dataset that could serve as a universal reference for benchmarking human embryo models remains unavailable^12–17^. Here, we developed such a human embryo development reference dataset through integration of the transcriptome data from six publicly available human datasets covering developmental stages from the zygote to the gastrula. Lineage annotations are contrasted and validated with corresponding available human and nonhuman primate datasets. Using this comprehensive and integrated reference dataset, we performed detailed comparisons with recently reported human embryo models, which revealed the risk of misannotation of cell lineages in embryo models when relevant human embryo references, such as the one developed in this work, were not utilized for benchmarking and authentication. To make our integrated reference dataset available for the public, we further developed a robust, user-friendly online early embryogenesis prediction tool, which can be utilized for benchmarking stem cell-based embryo models and human embryo-derived datasets. Further, we have also created two Shiny interfaces for convenient exploration of our reference datasets as well as primate comparative studies.

## Results

### Establish human embryo reference from zygote to gastrula

**Fig. 1:**
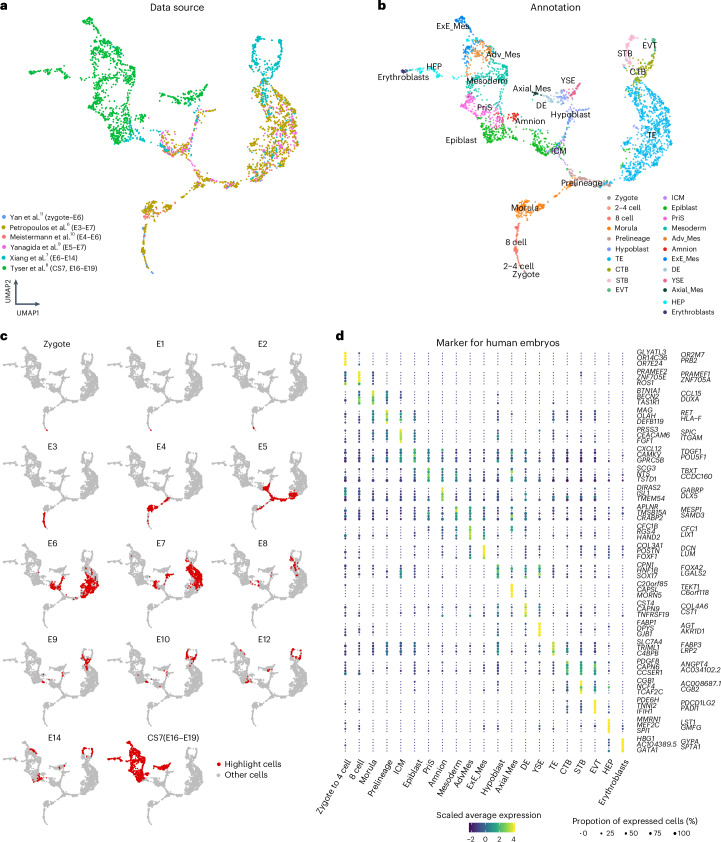
Construction of a human embryonic reference from zygote to the gastrula. **a**, A UMAP projection of the integration of six embryonic datasets. The color of each data point represents the source of the data. **b**, Similar to **a**, but the color indicates the cell annotations retrieved from each publication. **c**, Cells from different embryonic time points are highlighted on the human embryonic reference. **d**, A dot plot illustrating the expression of the top five lineage-specific genes used in the human embryonic reference. The size and colors of dots indicate the proportion of cells expressing the corresponding genes and scaled values of log-transformed expression, respectively. Source data

**Extended Data Fig. 1:**
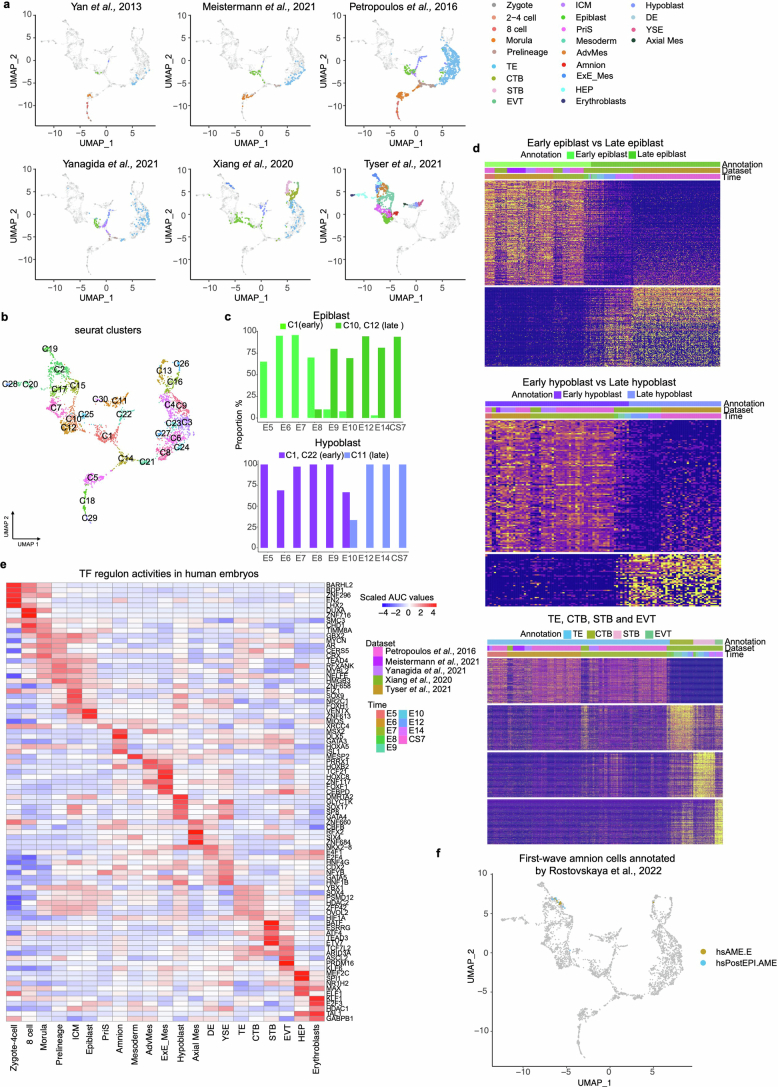
Clusters and regulon activity within the human embryonic reference. **a**, UMAP projection used in Fig. 1a shown by each embryonic dataset separately, colour of each data point indicates the cell annotations. **b**, Unassigned cluster distribution of UMAP used in Fig. 1a. **c**, Cell distribution in clusters for epiblast and hypoblast cells. **d**, Heatmap showing expression of DEGs between early and late epiblast, early and late hypoblast and DEGs among TE, CTB, STB and EVT. **e**, Heatmap displaying average AUC values of top 5 enriched regulons within each lineage. **f**, Highlighted first-wave amnion cells from Xiang et al. 2020 (based on the annotation from Rostovskaya et al., 2022). Abbreviations, hsPostEPI-AME: intermediates between epiblast and amnion cells; hsAME-E: early amniotic epithelium. Source data

**Extended Data Fig. 2:**
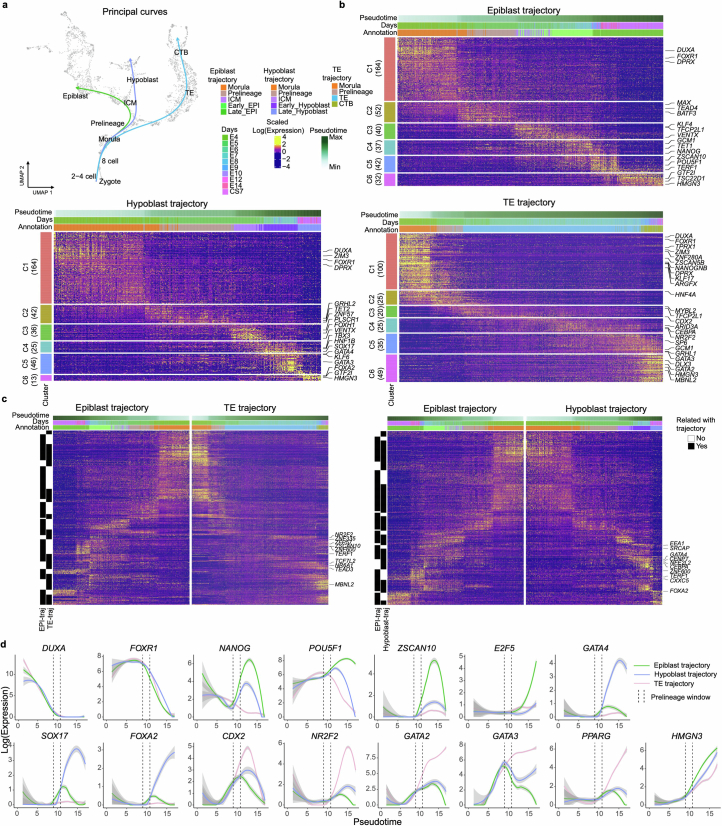
Transcription factor gene expression along the epiblast, hypoblast, and TE trajectories. **a**, Principle curves, and trajectories constructed from slingshot. **b**, Heatmap of expression of transcription factor (TF) genes which were significantly related to trajectories pseudotime. Cluster pattern of expression was indicated on the left with numbers indicating the number of TF genes. **c**, Joint heatmap showing expression of TF genes related to epiblast/TE trajectories and epiblast/hypoblast trajectories. The black and white annotation on the left indicated whether corresponding TF were significantly related to pseudotime. **d**, Expression dynamics (pseudotime) of selected transcriptional factor genes along three main trajectories. The confidence interval (error bands, 95%) is indicated by bandwidth. The measure of center and confidence intervals were calculated using the ‘loess’ function with default parameters in R software. Different trajectories are indicated by colours, respectively. Source data

**Extended Data Fig. 3:**
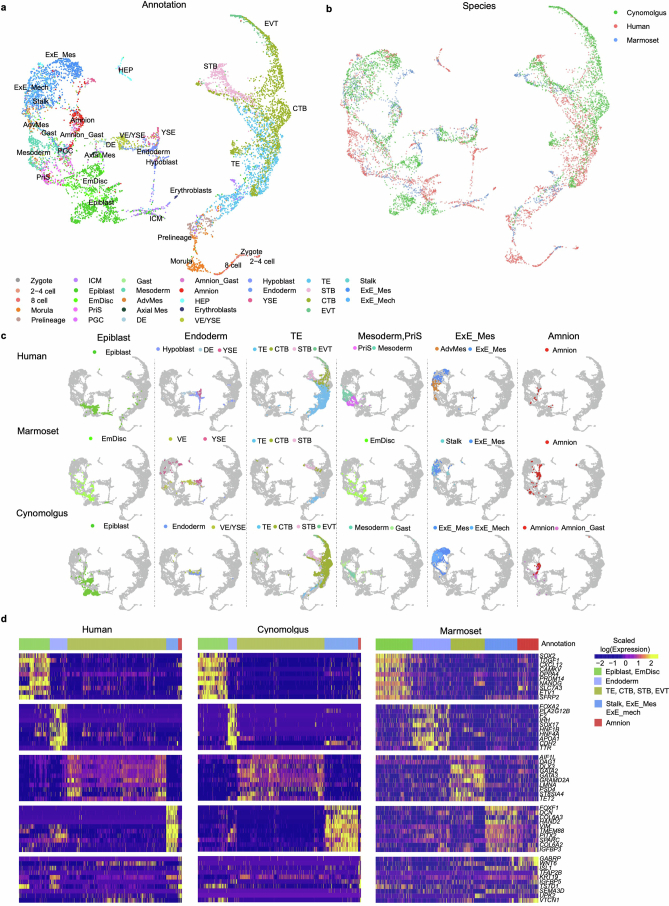
Cross-species integration involving cells from early human, cynomolgus monkey, and marmoset embryos. **a**, UMAP projection of the integrated datasets from six human, three cynomolgus monkey, and two marmoset embryos. Each data point’s colour corresponds to the cell annotations retrieved from each publication. **b**, Similar to (**a**), but the colour represents the species of the data. **c**, Highlights cells from each lineage belonging to their respective species in the cross-species integration. **d**, Expression of the top 10 lineage marker genes conserved in primate species. Abbreviations, ICM: inner cell mass; TE: trophectoderm; CTB: Cytotrophoblast; STB: Syncytiotrophoblast; EVT: Extravillous trophoblast; PriS: primitive streak; AdvMes: advanced mesoderm; DE: definitive endoderm; ExE_Mes:extraembryonic mesoderm; YSE: yolk sac endoderm; HEP: haemato-endothelial progenitors; EmDisc: embryonic disc; VE: visceral endoderm; SYS: secondary yolk sac; Gast: ‘Gastrula’; ExE_mech: extraembryonic mesenchyme. Source data

To create a human embryogenesis transcriptome reference covering these developmental stages, we collected six published datasets generated with scRNA-seq. We reprocessed these datasets, including mapping and feature counting, using the same genome reference (v.3.0.0, GRCh38) and annotation through a standardized processing pipeline (Methods). This approach was adopted to minimize potential batch effects as much as possible. These datasets include cultured human preimplantation stage embryos, three-dimensional (3D) cultured postimplantation blastocysts and a Carnegie stage (CS) 7 human gastrula at embryonic day (E) 16–19 isolated in vivo^6–11^ (Fig. 1a). For integration of these datasets, we employed fast mutual nearest neighbor (fastMNN) methods^18^ to establish a high-resolution transcriptomic roadmap. In total, expression profiles of 3,304 early human embryonic cells were embedded into the same two-dimensional (2D) space (Fig. 1a, Extended Data Fig. 1a and Supplementary Data 1). Considering both the published and updated cell type annotations, the resulting Uniform Manifold Approximation and Projection (UMAP) displays a continuous developmental progression with time and lineage specification and diversification (Fig. 1b,c). The first lineage branch point occurs as the inner cell mass (ICM) and trophectoderm (TE) cells diverge during E5, followed by the lineage bifurcation of ICM cells into the epiblast and hypoblast (Fig. 1c and Extended Data Fig. 2a)^6,10,19^. In the UMAP, early epiblast cells from E5 to E8 cluster together, whereas the majority of epiblast cells from E9 to CS7 form a distinct cluster annotated as ‘late epiblast’ (Extended Data Fig. 1a–d). A similar transition was observed from early to late hypoblast, occurring around E10 (Extended Data Fig. 1a–d). The UMAP also reveals that following extended 3D culture of human blastocysts, the TE matures into cytotrophoblast (CTB), syncytiotrophoblast (STB) and extravillous trophoblast (EVT), consistent with original annotations by Xiang et al.^7^ (Fig. 1b,c and Extended Data Fig. 1a,d). Cell cluster annotation of the CS7 gastrula dataset in the UMAP also revealed further specification of the epiblast into the amnion, primitive streak (PriS), mesoderm and definitive endoderm (DE), together with extraembryonic lineages including yolk sac endoderm (YSE), extraembryonic mesoderm (ExE_Mes) and hematopoietic lineages (hemato-endothelial progenitors (HEP) and erythroblasts), in agreement with original annotations by Tyser et al.^8^ (Fig. 1b,c and Extended Data Figs. 1a and 3c). The amnion has been suggested to form in two distinct waves^14^. In addition to the amnion cells in the CS7 dataset^8^, an earlier wave was postulated to occur in extended culture of human blastocysts^7^. As the majority of those cells intermingle with advanced mesoderm (Adv_Mes) and ExE_Mes cells from CS7 gastrula in our atlas (Extended Data Fig. 1f), we do not annotate those as amnion in accordance with previous literature^7,20^.

We then performed single-cell regulatory network inference and clustering (SCENIC) analysis^21^ to explore the activities of different transcription factors based on mutual nearest neighbor (MNN)-corrected expression values of these transcription factors across different embryonic time points. This analysis captured some known transcription factors known to be important for different cell lineage development, thus confirming lineage identities and working as a complement to the similar analysis reported in Chen et al.^22^, Mole et al.^12^, Weatherbee et al.^13^ and Fernandez-Gallardo et al.^23^, which were performed only for certain cell lineages or developmental stages. For example, we observed signatures of important transcription factors such as *DUXA* in 8-cell lineages^10^, *VENTX* in the epiblast^24^, *OVOL2* in the TE, *TEAD3* in STB, *ISL1* in amnion^25^, *E2F3* in erythroblasts and *MESP2* in mesoderm^26^, while ExE_Mes is enriched in *HOXC8* signatures (Extended Data Fig. 1e).

Slingshot trajectory inference^27^ based on the 2D UMAP embeddings revealed three main trajectories related to the epiblast, hypoblast and TE lineage development starting from the zygote (Extended Data Fig. 2a). In the epiblast, hypoblast and TE trajectories, 367, 326 and 254 transcription factor genes, respectively, were identified to show modulated expression with inferred pseudotime (Extended Data Fig. 2b and Supplementary Data 2). Transcription factors such as *DUXA* and *FOXR1* exhibit high expression during morula stages but decrease their expression during the development of all three lineages (Extended Data Fig. 2b,d). For the epiblast developmental trajectory, pluripotency markers such as *NANOG* and *POU5F1* are expressed in the preimplantation epiblast and decrease their expression following implantation, whereas *HMGN3* shows upregulated expression at the postimplantation stages (Extended Data Fig. 2b–d). Along the hypoblast trajectory, *GATA4* and *SOX17* show early expression while *FOXA2* and *HMGN3* demonstrated increased expression in the later stages. Within the TE trajectory, *CDX2* and *NR2F2* show early expression while *GATA2*, *GATA3* and *PPARG* show increased expression during TE development to CTB (Extended Data Fig. 2b,d). Notably, *HMGN3* is also associated with the later stage of the TE trajectory in a similar manner as seen in the epiblast and hypoblast trajectories (Extended Data Fig. 2b and Supplementary Data 2), a pattern also observed in the nonhuman primate transcriptome datasets^25,28–31^. Comparing the epiblast with the TE trajectories, genes such as *ZSCAN10* and *NR2F2* are specifically associated with the epiblast and TE trajectories, respectively, as they segregate from each other. Comparing the epiblast with the hypoblast trajectories, genes such as *GATA4* are specifically associated with the hypoblast trajectory (Extended Data Fig. 2b,c and Supplementary Data 2). Together, these trajectory inference analyses provide useful information for further functional characterization of key transcription factors that may play roles in driving the differentiation of the three main lineages in early human development (Supplementary Data 2).

We next identified unique markers for each distinct cell cluster from the zygote to the gastrula, including the known expression of *DUXA* in morula^32,33^, *PRSS3* in ICM cells^19^, *TDGF1* and *POU5F1* in epiblast, *TBXT* in PriS cells, *ISL1* and *GABRP* in amnion^25,34^ and *LUM* and *POSTN* in ExE_Mes^35^ (Fig. 1d and Supplementary Data 3). In addition, we identified genes such as *RBP4* (ref. ^36^) and *AFP*^37^ that were specifically upregulated in YSE but not in the hypoblast or DE (Supplementary Fig. 1a). When comparing ExE_Mes with embryonic mesoderm, genes including *DCN*, *ANXA1* and *POSTN* are specifically expressed in ExE_Mes but not in embryonic mesoderm^35^. In contrast, *ZNF738*, *TUBB2B* and *NPY* are enriched in embryonic mesoderm compared with ExE_Mes (Supplementary Fig. 1b). Further examination of the four subpopulations of HEP based on the reference UMAP shows that hemogenic endothelium stretches from the ExE_Mes to the HEP clusters (Supplementary Fig. 1c).

Given the enrichment of key marker genes and transcription factor regulatory networks, we are confident that our embryonic reference provides reliable transcriptome profiles for each lineage present in early human embryo development included in those datasets.

### Integration with nonhuman primates

It should be acknowledged that the existing human embryo reference datasets are still limited, with data from one single in vivo gastrulating embryo^8^. We therefore compared the six human embryo datasets with additional datasets from cynomolgus macaque, encompassing transcriptome profiles for preimplantation embryos^29^, embryos collected during gastrulation^30^ and in vitro cultured postimplantation embryos^25^. Furthermore, two additional datasets from the marmoset were also included, comprising a transcriptome profile for preimplantation embryos^28^ and implanted marmoset embryos at CS5–7, including spatial information^31^. Considering the effective performance of the MNN method in removing batch effects among datasets, similar MNN expression correction was performed for datasets from each species to eliminate intraspecies batch differences. This was followed by canonical correlation analysis to simultaneously anchor all datasets^38^. Through these efforts, single-cell transcriptional profiles from 11 datasets across three primate species were successfully embedded into the same 2D UMAP space (Extended Data Fig. 3a,b). This integrated UMAP revealed a continuum of transcriptome state changes. Cell lineage annotations and associated developmental stages reported identified in the UMAP also matched those by the original publications (Extended Data Fig. 3a and Supplementary Fig. 2), thus confirming that intra/interspecies batch differences were effectively removed during the aforementioned process.

From the cross-species integration, we observed that lineage developments from the prelineage to ICM and TE, from the ICM to epiblast and hypoblast, from the epiblast to PriS, mesoderm and amnion, and from the TE to CTB, STB and EVT, were well conserved in all primate species (Extended Data Fig. 3a,c). Marmoset embryonic disc (EmDisc) cells overlap with late epiblast-related lineages of the CS7 human gastrula, including the ‘epiblast’, ‘PriS’, ‘mesoderm’ and E16–E17 epiblast cells in cynomolgus monkey (Extended Data Fig. 3a,c and Supplementary Fig. 2). Visceral endoderm (VE) and YSE populations overlap well between human and marmoset datasets (Extended Data Fig. 3a,c). Marmoset cells reported with uncertain identity in the original report among CS7 secondary yolk sac, ExE_Mes, matched with secondary yolk sac and amnion cells in our analysis^31^. ‘Gastrula’ (Gast) cells in cynomolgus monkey overlapped with corresponding cell clusters in the human gastrula, including the mesoderm and PriS cells. Some cells identified as ‘Gast’ cells mainly from E16 and E17 cynomolgus monkey are aligned to part of the human Adv_Mes (Extended Data Fig. 2a,c and Supplementary Fig. 2). The position of cells from ‘Amnion_Gast’ from cynomolgus monkey^30^ overlap with cells from the human dataset, validating their identity as true amnion cells^8^. In addition, we observed that extraembryonic cells (ExE_Mes cells, stalk and extraembryonic mesenchyme) formed large clusters. Human Adv_Mes cells were situated between and intermixed with the embryonic mesoderm cells and extraembryonic cells on the reference map as a result of transcriptional similarities. On the basis of this observation and further analysis, we annotated 53 Adv_Mes cells as ExE_Mes (‘Restoration of previous annotations for published datasets’ section).

Utilizing the comprehensive primate reference, we identified conserved markers for the major lineages, including well-known pluripotency markers such as *SOX2* and *NANOG* for the epiblast, *SOX17* and *FOXA2* for endoderm cells, *GATA2* and *GATA3* for TE and its derivatives, *DCN* and *VIM* for extraembryonic cells and *GABRP* and *ISL1* for amnion cells (Extended Data Fig. 3d and Supplementary Data 4). Interestingly, some genes, such as the protein-coding gene *FAM124A*, whose function is uncertain, *MUSTN1*, which is related to the musculoskeletal system and normal embryo development^39^, *LIM2*, which encodes an eye lens-specific protein and *ADAM15*, which is involved in cell adhesion^40^, were identified as specific to human lineages but not to other nonhuman primates (Supplementary Fig. 3 and Supplementary Data 4). Thus, this comprehensive integrated scRNA-seq dataset of early primate embryos provides a framework for investigating the concordance and variance among different species. Importantly, nonhuman primate datasets provide further support to the annotations in our human embryogenesis reference.

### Stabilized query projection onto human embryonic reference

**Fig. 2:**
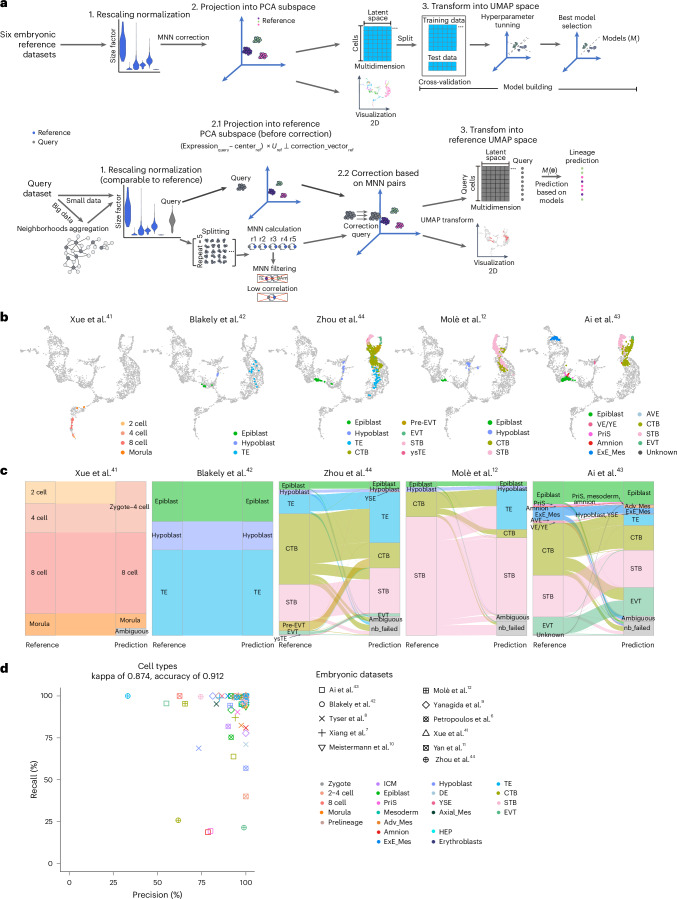
Validation of the early embryogenesis prediction tool. **a**, The processing workflow to project query cells onto the reference and cell type prediction. (1) Query data underwent rescaled normalization to ensure that expression values were comparable with the reference datasets. This step was taken after deciding whether to aggregate cells into neighborhoods for low-depth, large datasets. Cosine normalization of query expression involved removing the same grand center values from reference calculations and performing a dot product calculation with the left singular vectors (U) obtained from singular value decomposition during reference construction. (2) Orthogonalization removes variation along the reference batch correction vector, projecting onto the reference PCA subspace. Simultaneously, the query dataset was divided into smaller samples consisting of 200 cells, repeated five times, to calculate MNN pairs with the reference datasets separately. Uncertain MNN pairs were removed. Using the filtered MNN pairs, a batch correction vector was computed to correct the PCA coordination of the query dataset. (3) UMAP embedding was transformed using the UMAP model calculated from the reference (ref) dataset. Cell identities were predicted using the SVM models trained on the reference datasets within the same latent space after UMAP transformation. TE, trophectoderm; Am, amnion; r, repeat times. **b**, Projection of five embryonic datasets onto the human embryonic reference. The color represents the cell annotations for each publication. The light-gray points represent cells used in embryonic reference construction. **c**, An alluvial plot comparing the original cell type to the predicted identities from the early embryogenesis prediction tool. Predictions identified as ‘ambiguous’ or ‘nb_failed’ represent cells with uncertain predictions or cells that fail to form neighborhoods, respectively. **d**, Prediction precision and recall ratio for each cell type in the embryonic datasets. The shape and color indicate queried cell types and data sources, respectively. ysTE, yolk sac TE; VE/YE, visceral/yolk endoderm; AVE, anterior VE. Source data

**Extended Data Fig. 4:**
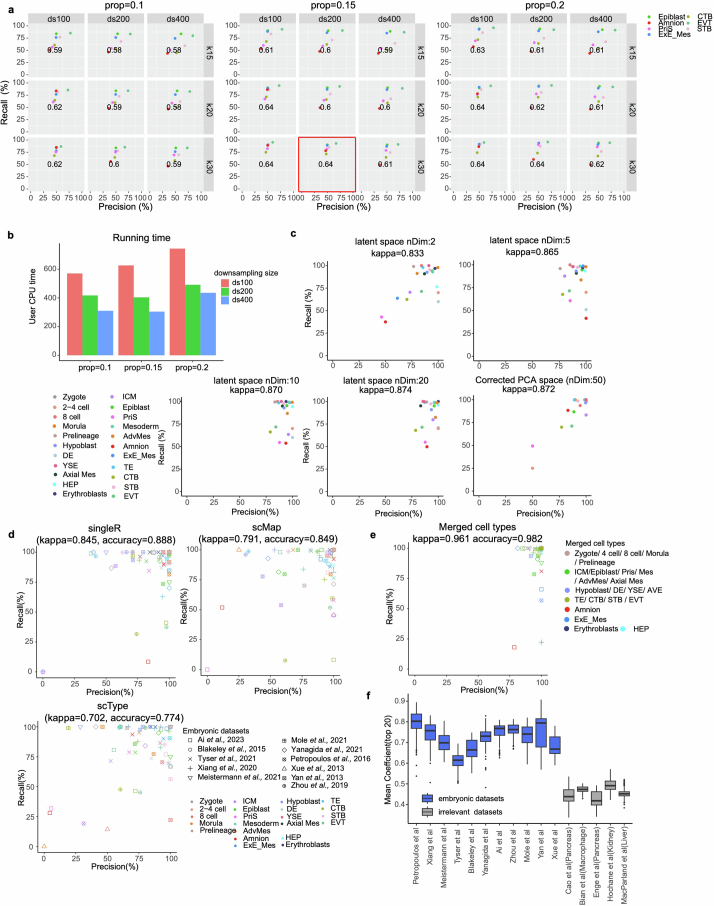
Parameter selection for processing workflow. **a**. Precision and recall ratios for aligned MNN pairs between the Ai et al., 2023 embryonic datasets and reference embryonic datasets under different parameters, including ‘prop’ (the proportion of randomly sampling during neighbourhood aggregation), ‘K’ (the number of neighbours considered), and downsampling size. The F-score values are shown on the plot. The colour of each data point represents the cell annotations from the Ai et al., 2023 embryonic dataset. **b**. CPU running time for MNN calculation under different ‘prop’ values and downsampling sizes. Here, ‘K’ was arbitrarily set to 30 as we determined this value had minimal influence on running time. **c**. Prediction precision and recall ratios for each cell type from all embryonic datasets using models trained with different numbers of dimensions. The colour of each data point represents the cell types. Performance metrics for the same cell types from different embryonic datasets were averaged. **d**. Prediction precision and recall ratios for each cell type using SingleR, scMap, and ScType. **e**, Prediction precision and recall ratio for merged cell types in the embryonic datasets. The shape and colour of data points indicate queried cell types and data sources, respectively. **f**. Shown the top 20 mean correlation coefficients of each cell from each dataset. The embryonic datasets are coloured blue, while irrelevant datasets are coloured grey. The boxplot rectangles represent the first and third quartiles, with whiskers extending 1.5 times the interquartile range above and below the box. A horizontal line inside the box indicates the median value. Outliers are indicated as dots. Source data

Having characterized the assembled human embryo reference dataset, we next sought to use this to establish a stabilized UMAP for evaluating the validity of stem cell-based embryo models. To achieve this, we distilled the entire fastMNN reference construction process into three major parts: (1) rescaling normalization, (2) principal component analysis (PCA) subspace projection after MNN correction and (3) UMAP projection (Fig. 2a). Throughout this analysis, adhering carefully to the assumptions that MNN pairs define the most similar cells of the same type across batches^18^, we divided the query data into small samples and filtered ambiguous pairs (Extended Data Fig. 4a,b and Methods). After generating comparable normalized data for the query dataset, it was projected onto the same PCA subspace and subsequently corrected in accordance with Haghverdi et al.^18^, followed by projection onto the same UMAP space as the reference (Fig. 2a). In addition, we trained support vector machine (SVM) classifier models for each lineage of reference cells in a 20-dimensional latent space, optimizing the hyperparameters for each model. Once the query cells were successfully transformed into the same latent space as the reference, their identities were predicted using these pretrained reference SVM models (Fig. 2a and Extended Data Fig. 4c). Query cells that did not correspond to those in our reference were filtered out based on correlation filtering and annotated as ‘nonrelated’ (Extended Data Fig. 4f and Methods).

To test the prediction performance and determine the best parameters for our prediction tool, entitled the early embryogenesis prediction tool, five additional embryonic datasets were utilized, including a prelineage embryo dataset spanning from the 2-cell to morula stage^41^, a blastocyst dataset^42^ and three peri-implantation and postimplantation datasets^12,43,44^ (Fig. 2b,c). Of note, regardless of the library preparation utilized to generate these datasets (for example, Smart-Seq2, Trio-seq or 10x sequencing) or whether the datasets were used as processed by the authors or reprocessed in-house, cell clusters in all five datasets displayed good alignments with their counterparts in our human embryo development reference dataset (Fig. 2b,c and Supplementary Fig. 4). Overall, we achieved a 0.961 kappa value and 0.982 accuracy when comparing published annotations of the main lineages during human embryo development with our predictions (Extended Data Fig. 4e). When considering a more granular classification of sublineages, we obtained a 0.874 kappa value and 0.912 accuracy (Fig. 2d). In addition, our prediction tool performed better than current methods such as SingleR^45^, scMap^46^ and scType^47^ in classification metrics, including kappa score and accuracy (Extended Data Fig. 4d). Our tool demonstrated robust prediction power for all lineages across all embryonic datasets (Fig. 2d and Extended Data Fig. 4d). Thus we conclude that this predictive pipeline delivers a highly accurate performance for scRNA-seq datasets, irrespective of upstream library preparation or data processing, which can be leveraged for benchmarking stem cell-derived embryo models and newly generated embryonic datasets.

### Mapping stem cells and derived lineages

**Fig. 3:**
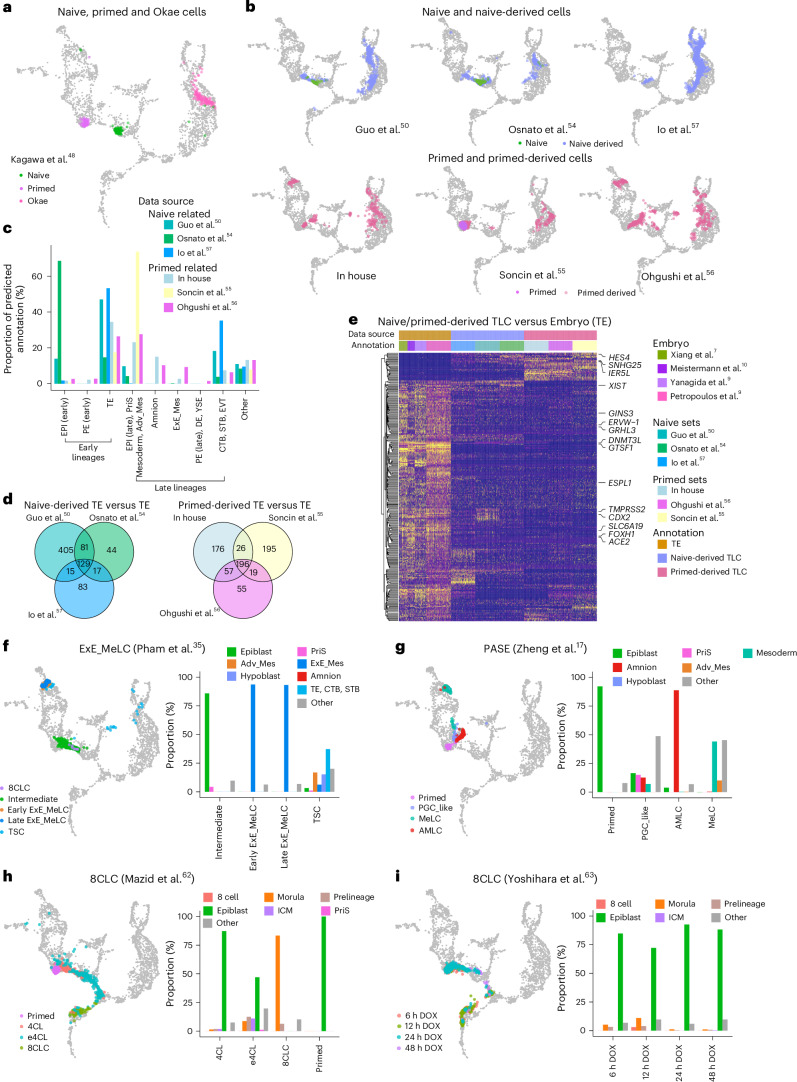
Application of the early embryogenesis prediction tool on stem cell models. **a**, The projection of naive, primed and Okae cells from Kagawa et al.^48^ onto the reference. The color of each data point represent the cell identity and gray cells the reference. **b**, The projection of naive or primed hPS cell-derived TLCs. **c**, A bar plot showing the proportion of predicted cell identities for naive and primed hPS cell-derived cells. **d**, A Venn diagram showing the overlap of DEGs between naive or primed-derived preimplantation TLCs and embryonic preimplantation TE cells. **e**, A heat map showing the expression of DEGs in preimplantation TLCs and embryonic preimplantation TE cells. The DEGs were conserved in all three naive hPS cell-derived TLC comparisons or conserved in all three primed hPS cell-derived TLC comparisons, primed hPS cell-derived TLCs and embryonic TE cells. **f**,**g**, The projection of cells (neighborhood nodes) from two studies modeling ExE_Mes cells and PASE. A bar plot showing the proportion of predicted cell identities stratified by cell types or time point. **h**,**i**, The projection of cells (neighborhood nodes) from two studies modeling 8CLCs. A bar plot showing the proportion of predicted cell identities stratified by cell original annotation. EPI, epiblast; PE, primitive endoderm; TSC, trophoblast stem cells; ExE_MeLC, extraembryonic mesoderm-like cell; PGC_like, PGC-like cell; MeLC, mesoderm-like cell; AMLC, amnion-like cells; 4CL, 4 chemicals + leukemia inhibitory factor (LIF) medium; e4CL, enhanced 4CL medium; DOX, doxycycline. Source data

**Extended Data Fig. 5:**
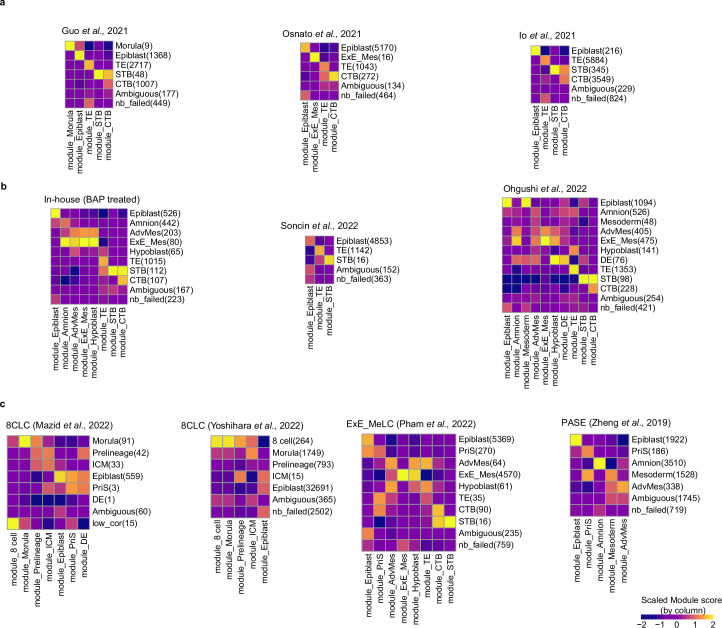
Module scores of cell models. Module scores of corresponding predicted lineages in three naïve stem cells models (**a**), three primed stem cells models **(b**), two 8-cell-like models, one extraembryonic mesoderm-cell-like model, and PASE models (**c**). Columns represent different lineage models scores, and rows represent predicted lineages for each dataset. The predicted cell numbers are included in parentheses.

Naive and primed human pluripotent stem (hPS) cells are considered analogous to the embryonic epiblast at preimplantation and perigastrulation stages, respectively. As expected, naive hPS cells were mapped to early epiblast cells before E9, whereas primed cells were projected to late epiblast cells (Figs. 1c and 3a and Extended Data Fig. 1c). Trophoblast stem cells, derived from blastoids using Okae et al. culture conditions^48,49^, mapped to E7–E10 TE and CTB cells, confirming their trophoblast identity. There is a longstanding debate whether human naive and primed hPS cells can be converted to trophoblast lineages^5,50,51^. One concern has been that converted putative trophoblast cells instead may be misannotated ExE_Mes or amnion lineage as some lineage markers are shared between these lineages^52,53^. For these reasons, we assessed the transcriptional profiles of published TE-like cells (TLCs) derived from naive and primed hPS cells, in addition to our own unpublished dataset of primed hPS cell-derived TLC (in-house) (Fig. 3b)^50,54–57^. Both naive and primed hPS cell-derived TLC were predicted as TE cells of the blastocyst or later trophoblast lineages. However, primed hPS cell-derived TLC also contained notable populations of both amnion and ExE_Mes cells (Fig. 3c). These predictions were confirmed by module score calculation based on lineage marker genes (Extended Data Fig. 5a,b).

**Extended Data Fig. 6:**
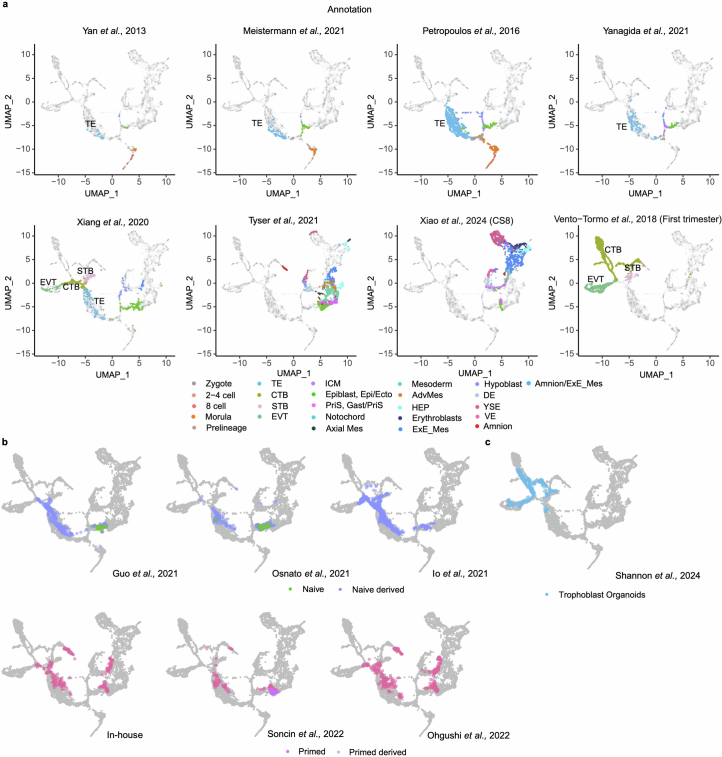
Extension of the human embryonic reference. **a**, UMAP projection of the integration of eight embryonic datasets including original six embryonic datasets used in Fig. 1, spatial transcriptomics from a Carnegie stage (CS) 8 human embryo (Xiao et al., 2024) and 10X-sequenced single-cell transcriptomes of STB, EVT, and villous CTB from first-trimester placentas (Vento-Tormo et al., 2018). Colour of each data point indicates the cell annotations. **b**, Projection of six datasets that use naïve or primed cells to model TLCs. The colour of each data point represents whether the cells (neighbourhood nodes) are naïve, primed, or their derived cells. **c**. Projection of organoids derived from the first trimester (Shannon et al., 2024). Source data

It has been suggested that human trophoblast stem cells are more similar to postimplantation trophoblast lineage or even first-trimester placental cells^22,58,59^. Since our reference atlas does not include such later stage cells, we decided to include two additional datasets to our existing atlas (Methods). One dataset is from recently published spatial transcriptomics of a CS8 human embryo^60^ and the other comprises 10x-sequenced single-cell transcriptomes of STB, EVT and villous CTB from first-trimester placentas^61^ (Extended Data Fig. 6a). According to this extended reference, CTB from first-trimester placentas clustered distinctly from CTB of peri-implantation embryos, whereas STB and EVT in first-trimester placentas and peri-implantation embryos were more closely related (Extended Data Fig. 6a). Using the same projection strategy as above, we projected the six datasets of human trophoblast stem cells derived from naive and primed hPS cells and an additional dataset from organoids derived from the first-trimester placenta^59^ onto this extended reference. TLCs derived from naive and primed hPS cells projected to pre- and peri-implantation TE lineage cells. In contrast, organoid cells derived from the first-trimester placenta projected more closely to first-trimester placenta cells (Extended Data Fig. 6b). Therefore, we conclude that TLCs derived from naive and primed hPS cells are more similar to pre- and peri-implantation TE lineages rather than to first-trimester placenta cells.

Among the 705 differentially expressed genes (DEGs) between naive and primed hPS cells, 32, 47 and 51 were also differentially expressed in the amnion, ExE_Mes and PriS, respectively, when compared with preimplantation TE cells (Supplementary Fig. 5 and Supplementary Data 5). Interestingly, DEGs highly expressed in amnion, ExE_Mes and PriS were also expressed specifically in the primed hPS cells (18 out of 18 genes). Conversely, genes preferentially expressed in TE cells were also enriched in naive hPS cells (49 out of 56 genes) (Supplementary Fig. 5d–g and Supplementary Data 5). The genes shared by naive hPS cells and TE cells, including transcriptional factors *NLRP2*, *NLRP7*, *TFAP2C*, *KLF4* and *ELF3*, may facilitate naive hPS cells to adopt a TE fate over alternative fates including amnion, ExE_Mes and PriS (Supplementary Data 5). We next compared the TLCs generated from both naive and primed hPS cells with the preimplantation TEs. We identified 129 and 196 DEGs consistently differentially expressed in naive or primed hPS cell-derived TLCs when compared with TE cells (Fig. 3d and Supplementary Data 6). Seventy-eight of these DEGs were shared in both naive and primed hPS cell-derived TLCs (Fig. 3e and Supplementary Data 6). TE transcription factors such *CDX2* and *GTSF1* were expressed at low levels in primed hPS cell-derived cells, while *HES4* (hes family bHLH transcription factor 4), which participates in transcriptional regulation and the Notch signaling pathway, was expressed only in stem cell-derived TLCs (Supplementary Fig. 6). *DNMT3L* involved in the process of DNA methylation was expressed at low levels in both naive and primed hPS cell-derived TLCs, and was particularly low in primed hPS cell-derived TLCs (Supplementary Fig. 6). *XIST* levels were also absent in primed hPS cell-derived TLCs, while some naive hPS cell-derived TLCs expressed significant levels of *XIST* (Fig. 3e and Supplementary Fig. 6), which suggests that primed hPS cell-derived TLCs might have an aberrant epigenetic state.

We next applied our reference map to evaluate published studies of differentiating naive and primed hPS cells into ExE_Mes and amnion, respectively^17,35^, and confirmed the accurate annotations of naive hPS cell-derived ExE_Mes cells as well as primed hPS cell-derived amnion in the stem cell-based, postimplantation amniotic sac embryoid (PASE) model (Fig. 3f,g). The PASE model also includes PriS- and mesoderm-like cells, which map as expected onto their in vivo counterparts of our reference map, while the primordial germ cell (PGC)-like cells developed in the PASE overlap with the PriS cells in our reference map. This observation suggests that our current reference can not readily resolve PGCs, as the original human gastrula dataset only included seven annotated PGCs, which is not enough to establish a discrete cluster on our reference UMAP.

There are recent studies reporting transient conversion of hPS cells into 8-cell-like cells (8CLC). Indeed, we observed a large proportion of morula-like cells but not 8CLC in Mazid et al.^62^, confirmed by module score calculation (Fig. 3h and Extended Data Fig. 5c). In ref. ^63^, we observed around 3.1% 8CLC 12 h after transient doxycycline treatment in doxycycline-inducible DUX4-TetOn hES cells, which is consistent with the data reported in the study (Fig. 3i).

### Evaluating the preimplantation blastoids models

**Fig. 4:**
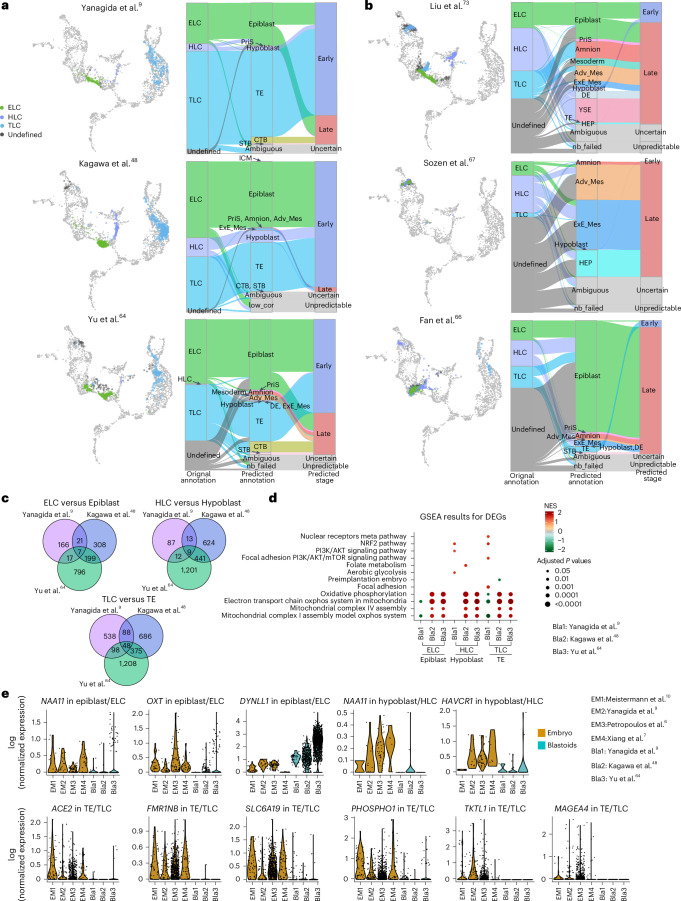
Application of early embryogenesis prediction tools on preimplantation blastoid models. **a**,**b**, The projection of blastoid cells (or neighborhood nodes) onto the human embryonic reference in naive-derived blastoids (**a**) and reprogrammed or EPS cell-derived blastoids (**b**). The color of each data point represents the cell annotations retrieved or restored for each publication. Light gray data points indicate cells used in embryonic reference construction. An alluvial plot comparing original cell-type annotations (ELC, HLC and TLC from the six blastoids) to the predicted identities obtained from the early embryogenesis prediction tool. **c**, A Venn diagram showing the overlaps of DEGs between blastoids with preimplantation embryonic lineages for three naive-derived blastoids. **d**, Selected significant Wikipathways demonstrating differences among the three naive-derived blastoids (Bla1–3) and preimplantation embryos from the embryonic reference, stratified by lineage. The colors indicate the normalized enrichment score (NES) and the size represents the Benjamini–Hochberg-adjusted *P* values from one-sided tests. **e**, Violin plots showing the expression of representative DEGs between blastoids (Bla1–3) and embryonic references (EM1–4). low_cor, low-correlation filtered. Source data

**Extended Data Fig. 7:**
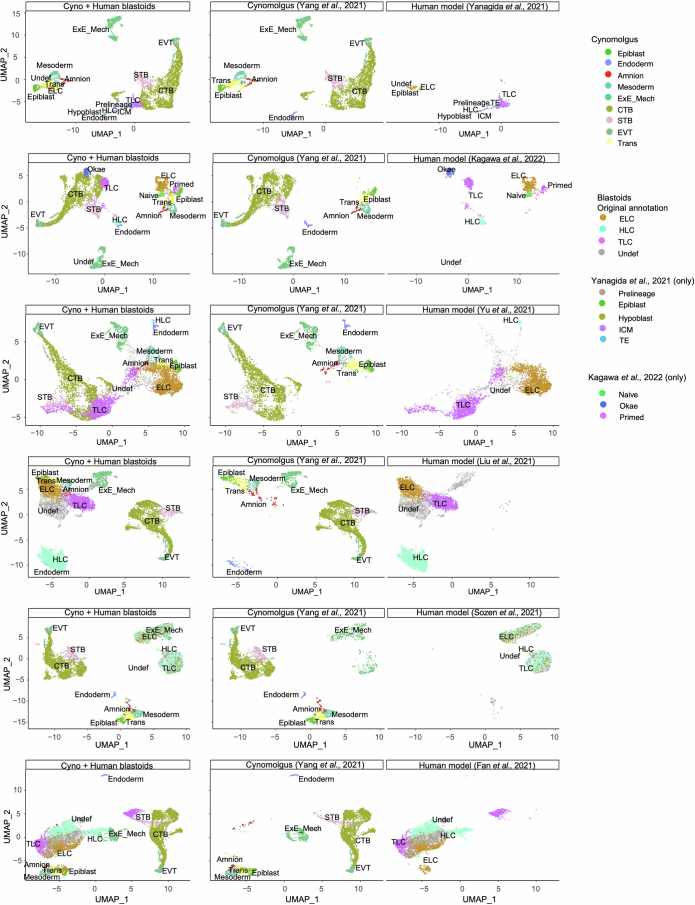
UMAP projection of Mutual Nearest Neighbours (MNN) cross-species integration of blastoid models and cynomolgus macaque at Day 14. Embryonic cells from Yanagida et al. and naïve, primed, and Okae cells from Kagawa et al. were also included in the integration. Overlay and separation by source of datasets is shown on the left and right, respectively. Colour corresponds to (Yang et al., 2021), coloured by cell type. Source data

**Extended Data Fig. 8:**
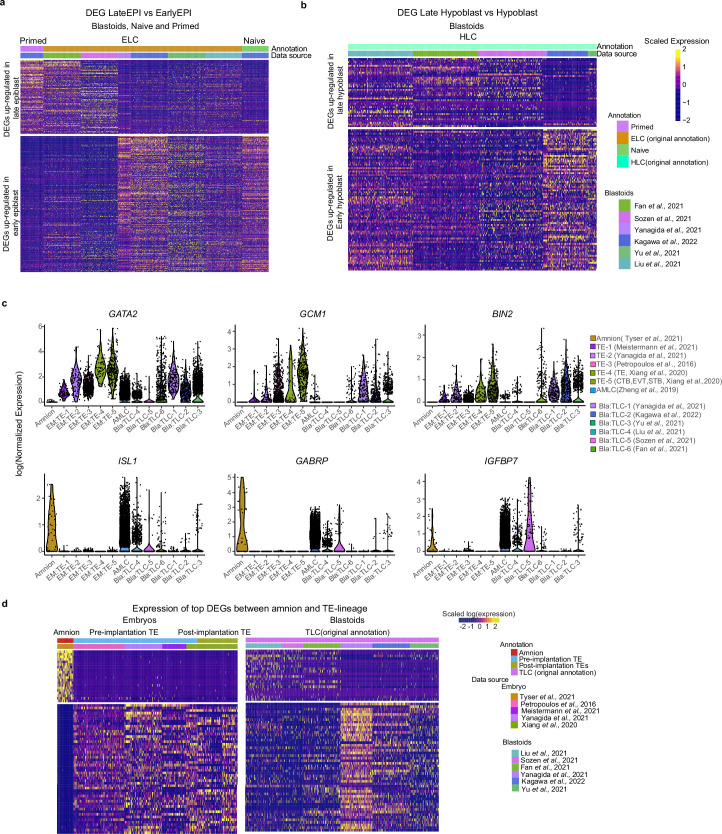
DEG between cell models and embryo cells. **a**, Heatmap displaying DEGs between early and late epiblast in primed cells, blastoids ELC cells (based on their original annotation), and naïve cells. **b**, Heatmap displaying DEGs between early and late hypoblast blastoids HLC cells (based on their original annotation). **c**, Violin plots showing log-transformed expression of key amnion and TE markers in amnion, TE, amnion-like cells (AMLC), and TLC from the six blastoids (based on their original annotation). **d**, Expression of DEGs between amnion and TE in embryonic amnion, TE, and TLC from the six blastoid models. For visualisation, cell types containing a large number of cells were randomly down-sampled to 200. Source data

**Extended Data Fig. 9:**
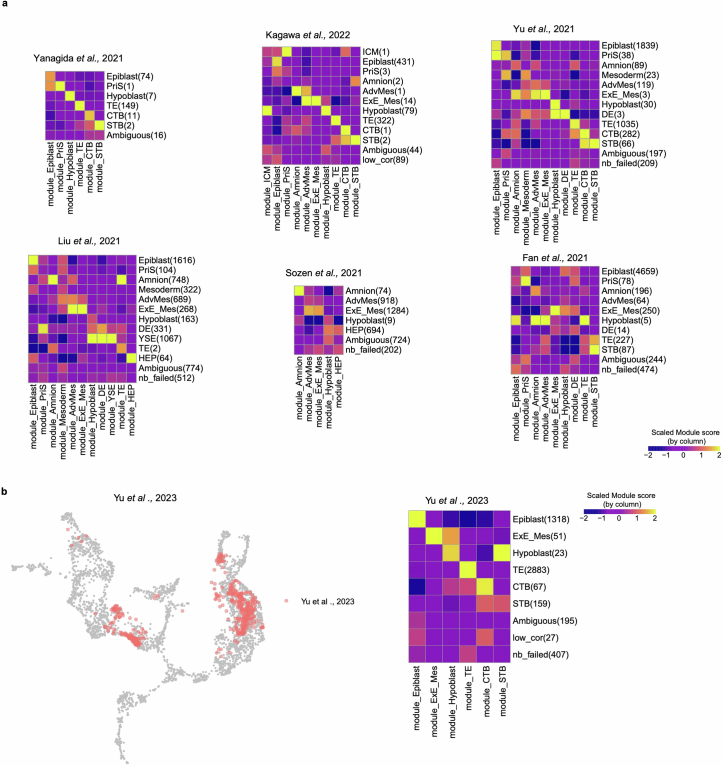
Module score validation of predicted lineages. **a**, Module score of corresponding predicted lineages in six blastoids. Columns represent different lineage models scores, and rows represent predicted lineages for each dataset. The predicted cell numbers are included in parentheses. **b**, Projection of most-recent blastoids from Yu et al., 2023 onto the human embryonic reference.

Next we explored the transcriptional profiles of blastoids established from naive hPS cells or extended pluripotent stem cells (EPS cells) or through partial reprogramming^9,16,48,64–67^. Projection of three naive hPS cell-derived blastoids reveals the presence of expected cell types, with the majority of annotated epiblast-like cells (ELC), hypoblast-like cells (HLC) and TLC overlapping with their counterparts in the human blastocyst, although a fraction of cells showed signatures more in line with postimplantation cell lineages (Fig. 4a,b and Extended Data Fig. 7). In addition, small fractions of cells with signatures resembling ExE_Mes and amnion were also detected in these naive hPS cell-derived blastoids (Fig. 4a). In blastoids generated from reprogramming of somatic human cells^65^, the majority of TLCs overlap with the amnion reference (Fig. 4b). Blastoids derived from EPS cells by Sozen et al.^67^ were reported to show blastocyst-like morphology but lack correct transcriptional profiles. In agreement with that finding, most cells in EPS cell-derived blastoids were predicted as ExE_Mes or Adv_Mes. EPS cell-derived blastoids from Fan et al.^66^ did contain TLCs, but the majority of the cells in the blastoids resembled ‘late epiblast’ cells (after E9) with very few HLCs (Fig. 4b). To validate these discrepancies in cell lineage composition in an independent manner, we further utilized published cynomolgus data^25^, which includes both amnion and trophoblast cells within the same dataset. Comparative transcriptome analysis of blastoids using cynomolgus data as a reference clearly shows that the majority of TLCs in blastoids of Liu et al.^65^ cluster with the amnion rather than the TE lineage (Extended Data Fig. 7). The majority of TLCs in blastoids from Sozen et al.^67^ are closely related to ExE_Mech (Extended Data Fig. 7). HLCs in the blastoids from Fan et al.^66^ did not align with the reference endoderm cells. Additionally, TLCs in blastoids from Liu et al. lack or express low levels of TE markers such as *GATA2*, *GCM1* and *BIN2*, but instead express amnion markers *ISL1*, *GABRP* and *IGFBP7* (Extended Data Fig. 8c,d). Furthermore, we utilized DEGs between early and late stages of the epiblast and hypoblast cells (Extended Data Fig. 1d and Supplementary Data 7) and checked their expression in blastoids (Extended Data Fig. 8a,b). The ELCs in EPS cell-derived blastoids^66,67^ preferentially express late epiblast DEGs but low expression levels of early epiblast DEGs, similar to primed hPS cells (Extended Data Fig. 8a). HLC of blastoids generated through partial reprogramming^65^ or using EPS cells^66,67^ expressed DEGs enriched in late hypoblast cells. Predicted annotations were further justified by module score calculation (Extended Data Fig. 9). Together, our analysis supports that naive hPS cell-derived blastoids are composed of cells with transcriptional profiles in line with those in human blastocysts. However, detailed analysis could still identify differences in gene expression between these naive hPS cell-derived blastoids and the human reference. On the basis of the Gene Set Enrichment Analysis (GSEA)^68^ for each comparison within each lineage, pathways related to energy production, including ‘oxidative phosphorylation’ and ‘electron transport chain oxphos system in mitochondria’, were upregulated in Kagawa et al.^48^ and Yu et al.^64^ but not in Yanagida et al.^9^ Metabolic rewiring has been suggested to be important for blastoid formation^69^. Genes related to ‘focal adhesion’ and the ‘focal adhesion PI3K/AKT/mTOR signaling pathway’, which are important for cell attachment and migration, were instead only upregulated in Yanagida et al.^9^ (Fig. 4d and Supplementary Data 8). Additionally, there were 7, 9 and 48 DEGs shared by all three naive hPS cell-derived blastoids when compared with the human reference (Fig. 4c and Supplementary Data 8). Specifically, in naive hPS cell-derived blastoids, *NAA11* showed reduced expression in ELC and HLC. *OTX and HAVCR1* were also poorly expressed in ELC and HLC, respectively. Expression of *ACE2*, *FMR1NB*, *SLC6A19*, *PHOSPHO1*, *TKTL1* and *MAGEA4* were also lacking in TLC of naive hPS cell-derived blastoids (Fig. 4e). It is unknown whether these DEGs translate into functional differences but such aberrant expressions should be taken into consideration when using these models. One recent example is modeling of severe acute respiratory syndrome coronavirus 2 (SARS-CoV-2) placenta infection using stem cell models. SARS-CoV-2 infects cells via its spike protein binding to the host entry receptor angiotensin-converting enzyme 2 (*ACE2*). Several studies have examined SARS-CoV-2 infection and *ACE2* expression in trophoblast organoids and in alignment with our analysis of blastoids, they also have seen reduction of *ACE2* levels and SARS-CoV-2 infection in such in vitro models, highlighting important functional differences between stem cell models and embryonic cells^70,71^.

### Evaluating stem cell-derived postimplantation embryo models

**Fig. 5:**
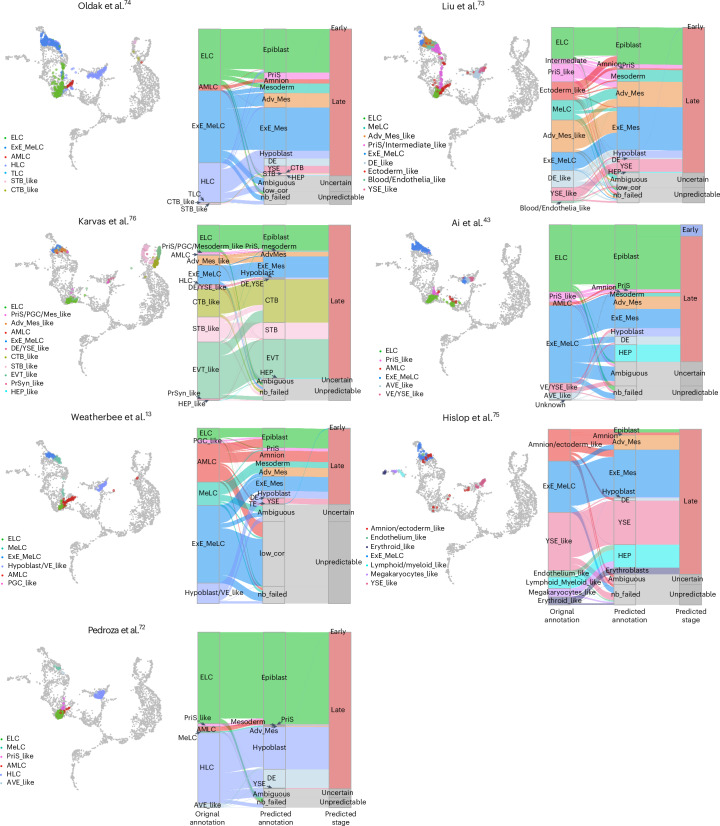
Application of early embryogenesis prediction tools on postimplantation blastoid models. The projection of blastoid cells (or neighborhood nodes) onto the human embryonic reference (left side of each reference image). The color of each data point represents the cell annotations retrieved or restored for each publication. The light gray data points indicate cells used in embryonic reference construction (right side of each reference image). The alluvial plots compare original cell-type annotations to the predicted identities obtained from the early embryogenesis prediction tool. ExE_MeLC, extraembryonic mesoderm-like cell; PGC_like, PGC-like cell; MeLC, mesoderm-like cell; AMLC, amnion-like cells; STB_like, STB-like cells; CTB_like, CTB-like cells; YSE_like, YSE-like cell; EVT, EVT-like cell; HEP_like, HEP-like cell; PriS_like, PriS-like cell; AVE_like, anterior VE-like cell; VE, VE-like cell; PrSyn_like, primitive syncytium-like cell; DE_like, definitive endoderm-like cells; AdvMes_like, advanced mesoderm-like cells; Blood/Endothelia_like, blood/endothelia-like cells; PriS/Intermediate like, primitive streak/intermediate-like cells; Ectoderm_like, ectoderm-like cells. Source data

**Extended Data Fig. 10:**
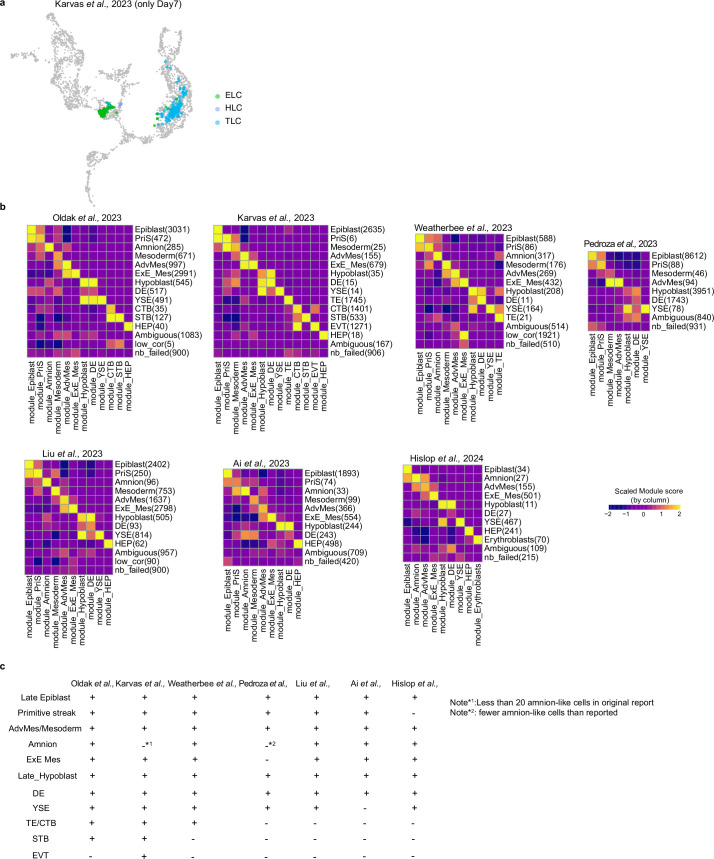
Analysis of post-implantation models. **a**, Highlighted cells from the Day 7 blastoids (neighbourhood nodes) from Kavas et al., 2023. The colour of each data point represents the cell annotations retrieved from original publication. **b**, Module score of corresponding predicted lineages in seven post-implantation models. **c**, Presence of post-implantation lineage-like cells in post-implantation embryo models.

Following implantation, the human embryo undergoes major organizational changes crucial for gastrulation and subsequent development. Several stem cell-derived, postimplantation models have been developed to mimic the development of human embryos during this window. In addition to capturing morphogenesis and structural similarities to human postimplantation embryos, it is important to understand which lineages they are composed of. To address this, we projected recent studies^13,43,71–75^ onto our human embryogenesis reference to evaluate their similarities with postimplantation lineages (Fig. 5 and Extended Data Figs. 10a,b). Three studies^43,72,73^ combine pluripotent cells with hypoblast-induced cells, generating cells with signatures aligning with the embryogenesis reference cells including epiblast, PriS, mesoderm, ExE_Mes, amnion and late hypoblast (Fig. 5). Two other studies^13,74^ also included TLC in the assembloids, cultured in suspension, while the third started from naive-derived blastoids that were allowed to grow attached in 2D or imbedded in 3D^71^. The blastoid-based model had the best contribution to the TE compartment in the embryonic reference (Fig. 5 and Extended Data Fig. 10c), possibly due to the attachment strategy that might better support TE expansion. It is important to note that postimplantation models do not necessarily need to capture all lineages of the human embryo to be useful. This is exemplified in the recent study by Hislop et al.^75^, which modeled postimplantation development without a TE compartment but succeeded in establishing yolk sac hematopoiesis, which was evident also from mapping to the embryogenesis reference (Fig. 5). Further, our tool was unable to resolve amnion cells from Pedroza et al.^72^ and Karvas et al.^76^, possibly due to two distinct reasons. We observed that amnion-annotated cells from Pedroza et al.^72^ are projected together with epiblast and PriS. Using the published annotations and gene expression matrix from Pedroza et al.^72^, we identified that only 62 out of 460 amnion-annotated cells exhibited at least one read for any one of the amnion markers, *ISL1*, *GABRP*, *VIT*, *VTCN1*, *WNT4* or *WNT6*, which is insufficient to classify as amnion. It is still possible that there are early amnion cells in this dataset, which are lacking in the Tyser et al.^8^ reference. In the Karvas et al.^76^ study, less than 15 amnion cells are reported, which are interspersed with Adv_Mes-like cells^71^. In this case, the amnion cells are probably too few to form an independent amnion neighborhood, but instead mix with ExE_Mes cells in our reference map due to their shared transcriptional profiles.

### A web platform for embryonic study

During this study, we have curated a total of 41 processed datasets, including their raw expression data, original annotation and predicted annotation. Among these datasets, eight human embryonic datasets, six preimplantation blastoids datasets, one 8CLC dataset and one PASE dataset were reprocessed by us using the same mapping strategy (10x using Cell Ranger^77^ and non-10x using STAR^78^) and genome annotation^6–11,42,43,48,63–67^. This effort has allowed us to eliminate batch differences arising from different processing pipelines or gene annotations, providing a valuable resource for future investigations.

**Fig. 6:**
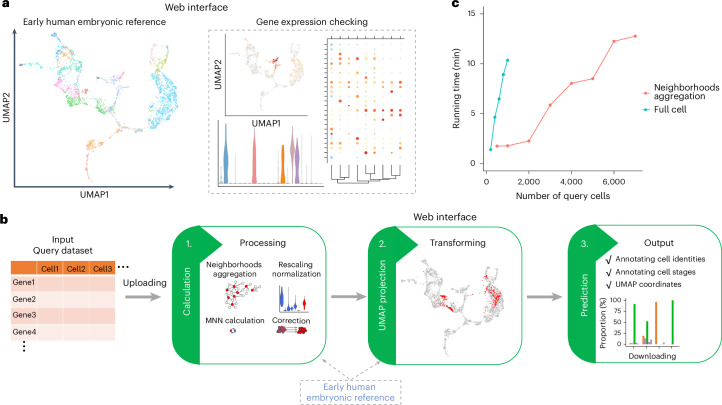
Web interface for our online resources. **a**, A schematic of the web interface for the human embryonic reference. **b**, A schematic of the web interface for the early embryogenesis prediction tool. **c**, The running time for webtool with different numbers of query cells. Source data

To facilitate comparative studies and easy access to analysis of single-cell datasets related to the developmental stages of zygote to gastrulation, we have developed an interactive online tool based on the human embryogenesis reference and the extended primate cross-species embryonic reference using ShinyCell^79^ (Fig. 6a). This can be used to browse the expression of selected genes in both the human and primate reference atlas. Furthermore, we have transformed the prediction pipeline into an online analytical application, which we entitled the early embryogenesis prediction tool (Fig. 6b). Our primary focus was to create a user-friendly interface, ensuring that researchers can directly access and utilize it by providing only the gene expression matrix. The entire process of normalization, projection and annotation takes less than 15 min for fewer than 7,000 cells (Fig. 6c). We envision broad usage of these assembled datasets and online tools for the authentication of human embryo models, in turn supporting the development and application of this emerging field.

## Discussion

In this Resource, we have integrated available human embryo datasets to create a transcriptional reference map of human embryonic development from zygote to gastrula. This assembled dataset will hopefully facilitate deeper understanding of the transcriptional networks that are operating and directing early human embryo development. The resource will also aid in validating and aligning stem cell-based embryo models with embryo equivalents, while also providing a transcriptional benchmark for the development of future models and optimization of stem cell cultures. The embryonic reference tool effectively distinguished early human embryo identities from different stages and lineages. We anticipate that the strategy we utilized for building the reference atlas, project and predict query datasets can be extended to other developmental stages, specific organs and other species, although this is outside the scope of the current study. The development of our reference depended on the quality of the datasets utilized and, as such, if datasets of lower quality or richness (for example, cell types lacking, insufficient number of cells, shallow read depth and so on) would be incorporated, it would result in reduced precision. That said, we have demonstrated the expandability of our embryonic reference, even when shallow sequenced datasets are utilized, though not ideal as they require additional processing by aggregation. An additional caveat we need to stress is that the incorporation spatial transcriptional analysis is not at a single-cell level, which impacts the utility of the cellular signatures as multiple cell types may be captured within the same area. In both instances, we see that the strategy used for the construction of our reference is robust. This strategy may also be helpful within the field of stem cell-based cell therapies to characterize the cellular composition of on- and off-target cell types^80^.

There are important limitations of our tool that should be considered when interpreting results obtained. First, an inherited limitation of MNN correction makes it challenging to correct datasets without shared similar identities. False-positive predictions may therefore occur for cells with transcriptome signatures that are not present in our reference but share close similarities. We partially resolved this issue by including correlation filtering in our processing pipeline. To test this we used unrelated datasets, including five public scRNA-seq datasets from human pancreas, macrophages, fetal kidney development and liver tissues (Methods) and determined that a median top correlation coefficient threshold of 0.5 can eliminate unrelated cell types. However, this is still an important aspect to bear in mind. Another limitation is that the embryonic reference dataset contains few PGCs, with similarities in gene expression to PriS, and could therefore not be resolved as a distinct cluster which means that we can not identify PGCs using our tool. This could be resolved in the future with additional in vivo reference datasets, ideally sequenced using Smart-seq based platforms to ensure richness of the reference. Distinguishing between ExE_Mes cells and embryonic mesoderm cells proved challenging due to shared gene expression similarities and lack of well defined markers. Cross-referencing with the spatial information provided by the marmoset dataset allowed us to reclassify some Adv_Mes cells as ExE_Mes cells. Genes specifically expressed in ExE_Mes cells, such as *DCN*, *ANXA1* and *POSTN*^35^, may be helpful in addressing these issues. Nonetheless, clear boundaries between these cell types remains a challenge and deeper understanding about these lineages are needed.

miloR is a tool designed for complex single-cell datasets^81^, which we utilized to form neighborhoods to aggregate gene expression of cells with similar gene expression signatures. Our previous analyses^74^ confirmed that neighborhoods were approximately homogeneous in their cell-type composition, providing improved prediction accuracy by addressing sparsity in 10x-type datasets through gene expression aggregation within the same neighborhood. While this aggregation method is beneficial for analyses with sparse datasets, it should be noted that a certain proportion of cells may fail to form neighborhoods (labeled as ‘nb_failed’ in prediction), leading to information loss for those cell types. Furthermore, if a query dataset contains a very rare subpopulation with limited number of cells, our tool may be unable to form independent neighborhoods of that rare subpopulation and instead merge those cells with the closest resembling cells. Indeed, this was observed with the few amnion cells in the Karvas et al. dataset^76^.

Our study has also provided insights into the complexity of stem cell states by comparing the transcriptional profiles of naive and primed hPS cells to early embryonic lineages. The mapping of naive cells to the early epiblast and primed cells to the late epiblast stages resonates with their developmental potential and distinct molecular characteristics. This alignment supports the notion that naive and primed hPS cells mimic distinct developmental time points. The longstanding debate over the ability of human naive and primed stem cells to transition into trophoblast lineages has also been addressed in our study, presenting a nuanced view of this complex process. The identification of trophoblast stem cells cultured under specific conditions mapping to the expected embryonic stages reinforces the notion that, with precise environmental cues, both primed and naive stem cells can be guided into specific lineage pathways. However, the notable presence of amnion and extraembryonic mesodermal cells in primed-derived cell populations underscores the intricate balance and potential for diverse differentiation outcomes inherent in these cells. This finding underscores the necessity for refined cellular markers and rigorous validation strategies to ensure accurate cell lineage fate identification. Although both naive and primed hPS cells can make TLCs, the transcriptional profiles of hPS cell-derived TLCs still differ from their in vivo counterparts. Further, our analysis identifies the same concern for current blastoids: even though they contain the expected cell types, the current blastoids show notable transcriptional differences compared with human blastocysts. From the primed hPS cell-derived TLCs, it appears that part of the problem is related to epigenetic dysregulation as these cells show reduced levels of DNA (cytosine-5)-methyltransferase 3-like (DNMT3L) and long noncoding RNA XIST, which mediated X-chromosome silencing. In this aspect, the naive hPS cell-derived TLCs are more alike the embryo trophoblast lineage. It will be interesting to examine whether starting from an even earlier stem cell state could give added value to embryo models. From that perspective, it is exciting to see that transient conversion to earlier states, such as the 8-cell or morula-like stages could be confirmed in our early embryogenesis prediction tool. We hope this tool will facilitate development of more refined systems to study early human embryogenesis and benchmark these against the human embryo. With the generation of additional in vivo datasets containing postimplantation embryonic or extraembryonic tissues, we aim to continue to expand this reference.

## Methods

### Ethics declarations

The research complies with all relevant ethical regulations. hES cell line HS975 was previously derived (Swedish Ethical Review Authority: 2011/745:31/3). Donors gave their informed consent for the derivation and subsequent use of the hES cell lines. No compensation was provided to the donating couples.

### Differentiation of primed-derived trophoblast-like cells

hES cells growing on hrLN-521 (10 μg ml^−1^; Biolamina, LN521-02) in NutriStem hPSC XF (Biological Industries, 05-100-1A) were enzymatically dissociated and seeded onto new plates also coated with hrLN-521 at a cell density of 1.84 × 104 cells cm^−2^. At 24 h after seeding, hPS cells were moved to a 5% CO_2_/5% O_2_ incubator and differentiated into trophoblastic cells using NutriStem hPSC XF without bFGF (basic fibroblast growth factor) and TGFb (transforming growth factor β) that was supplemented with 10 ng ml^−1^ BMP4 (bone morphogenetic protein 4) (R&D Systems, 314-BP-050e), 1 µM A83-01 (R&D Systems, 2939) and 0.1 µM PD173074 (Sigma-Aldrich, P2499) (BAP treated) according to a previously published protocol^82^. The medium was changed daily during the 4 days of differentiation.

### In-house library preparation for scRNA-seq

Cells were dissociated with TrypLE Select (4 min at 37 °C; Thermo Fisher Scientific, 12563011) and deactivated by adding NutriStem hPSC XF and collected in 0.04% BSA (Sigma-Aldrich, A7979) in PBS^−/−^ and used following the cell multiplexing oligo (CMO) labeling for scRNA-seq protocols with feature barcode technology protocol (10x Genomics, CG000391). Viable cells were counted using the NC-200 Nucleocounter (Chemometec) (four CMO samples combined, aiming for 2,000 cells per sample). Chromium Next GEM Single Cell 3′ Reagent Kits v.3.1 (Dual Index) with feature barcode technology for cell multiplexing (10x Genomics, CG000388) was used following the user guide. CMO libraries and transcriptome libraries were sequenced using Illumina NextSeq 2000.

### Preprocessing of human scRNA-seq data and gene expression quantification

In-house 10x Genomics multiplexed primed-derived trophoblast scRNA-seq data were processed using the ‘cellranger multi’ pipeline (v.6.1.1) with default parameters^77^. Published scRNA-seq data were processed using the ‘cellranger count’ pipeline (v.3.0.0). The STAR aligner (v.2.5.3b)^78^ was employed to map reads to the GRCh38 reference genome (v.3.0.0, GRCh38, downloaded from the 10x Genomics website). To minimize differences associated with the sequencing platform and data processing, published Smart-Seq2 datasets and Yan et al.^11^ were also remapped to the same reference using the same aligner with default settings. Only uniquely mapped reads were retained for gene expression quantification. Raw read counts were further estimated using rsem-calculate-expression from the RSEM tool (v.1.3.0), with the option of ‘--single-cell-prior’^83^. Datasets without a note indicating ‘reprocessed’ in Supplementary Data 9 were based on the previously processed expression matrix reported in their original publication.

### Preprocessing of scRNA-seq data and gene expression quantification for marmoset monkeys (*Callithrix jacchus*) and cynomolgus macaques (*Macaca fascicularis*)

The scRNA-seq transcriptomes of marmoset embryos were sourced from Boroviak et al.^28^ and Bergmann et al.^31^. Expression matrices were obtained from https://github.com/Boroviak-Lab/SpatialModelling. Cells derived from maternal material were excluded from the analysis. For cynomolgus macaques, embryonic scRNA-seq transcriptomes were downloaded from Nakamura et al.^29^, Ma et al.^30^ and Yang et al.^25^. Nakamura et al.^29^ data were reprocessed and remapped using STAR (v.2.5.3b) with the reference genome ‘Macaca_fascicularis_5.0.96’ from Ensembl^84^, which is used in Yang et al.^25^. Gene expression was quantified using RSEM (v.1.3.0) with cell annotations downloaded from the original publications. Ma et al.^30^ data were reprocessed and remapped using Drop-seq tool (v.2.5.1, https://github.com/broadinstitute/Drop-seq) with the same reference genome. Cell annotations for Yang et al.^25^ were downloaded from http://www.nhp-embryo.net, with raw reads kindly provided by the authors.

### Quality control and normalization

To filter out low-quality cells, we implemented cutoffs based on the number of expressed genes (nGene) and the percentage of mitochondrial genes (percent.mito). For Smart-Seq2 datasets, high-quality cells had at least 2,000 nGene and a percent.mito of less than 0.125. For 10x datasets and other datasets, the cutoffs were determined from original publication or in cases where too few nGene or too high percent.mito was observed, the cutoff was further determined based on the general distribution of nGene and percent.mito. An upper limit for the number of nGene was set to prevent doublets in 10x datasets. Detailed parameters used in quality control for each dataset are listed in the Supplementary Data 9.

After quality control and the exclusion of mitochondrial genes, genes with expression in at least five cells were selected and assessed separately for each dataset. Subsequently, we calculated log-normalized counts using the deconvolution strategy implemented by the ‘computeSumFactors’ function in the R scran package (v.1.14.6)^85^, followed by rescaled normalization using the ‘multiBatchNorm’ function in the R batchelor package (v.1.2.4)^18^. This ensured that the size factors were comparable across batches. The log-transformed normalized expression after rescaling was then utilized for human dataset integration, marker detection and identification of DEGs.

### Restoration of previous cell annotations for published datasets

Original cell identities for human embryonic datasets, excluding those from Petropoulos et al.^6^, Xiang et al.^7^ and Tyser et al.^8^, were obtained from their respective original publications. We utilized the most recently published annotation from Meistermann et al.^10^ for the Petropoulos et al.^6^ dataset in our analysis. In addition, we examined the ICM cells reported by Stirparo et al.^15^ These cells showed substantial overlap with the ICM cells reported by Yanagida et al.^9^ (Supplementary Fig. 7a). Specifically, 29 cells were clustered into cluster C1, exhibiting higher expression of ICM-specific markers identified by Radley et al.^19^ (Supplementary Fig. 7b,c). Consequently, these cells were classified as ICM cells in our analysis for Petropoulos et al.^6^. Using our embryonic reference, we observed misannotated cells from the Xiang et al.^7^ dataset, as also reported by Chhabra et al.^20^ (Supplementary Fig. 7d) and, as such, we used the annotations provided by Chhabra et al.^20^. In addition, we reclustered the amnion, PriS and epiblast cells from Tyser et al.^8^, as reported in Zheng et al.^34^. We examined the reported markers *ISL1*, *DLX5*, *CLDN10* and *TFAP2A* for amniotic ectoderm as identified from spatial sequencing of human gastrula in Xiao et al.^60^. Twenty cells that were previously annotated as either PriS (19) or epiblast (1), were reannotated as amnion cells because they highly expressed amniotic ectoderm markers but had low expression of *TBXT* or *POU5F1* (Supplementary Fig. 7e) (created by the Seurat pipeline with 2,000 top variable genes and 25 top principal components (PCs)). Further, the cross-species integration analysis performed in Yang et al.^25^ with the human CS7 data from Tyser et al.^8^ displayed that some of the human Adv_Mes cells overlapped with the cynomolgus monkey extraembryonic mesenchyme cells. In agreement with their observation, integration of the CS7 human gastrula together with postimplanted marmoset data^31^, we observed that a proportion (53 of 159 cells) of the previously annotated human Adv_Mes cells overlapped with the marmoset stalk cells. As such, 53 cells previously classified as Adv_Mes cells were reannotated as ‘extraembryonic mesoderm’ cells, using the spatial data provided for ‘Stalk cells’ from the postimplanted marmoset^31^ (Supplementary Fig. 7f) during cross-species integration (created by the ‘RunFastMNN’ function with 2,000 top variable genes and 25 top PCs). We then performed a DEG analysis between the remaining Adv_Mes cells and extraembryonic mesoderm cells and observed that the 53 cells displayed intermediate expression for both these lineages, providing additional evidence supporting the reannotation (Supplementary Fig. 7g). Additionally, a total of 330 EVT and 649 STB cells were identified from previously annotated CTB cells in the cynomolgus monkey (*Macaca fascicularis*)^25,29,30^, and 51 CTB and 77 STB cells in the marmoset^31^ and confirmed by the expression of markers from human CTB, STB and EVTs (Supplementary Fig. 8). TE cells from Ai et al.^43^ were selected using the Seurat pipeline (with 2,000 top variable genes and 25 top PCs) to further delineate the CTB, STB and EVT lineages. For datasets lacking cell annotations, we restored annotations based on their source code or descriptions provided in the original publication. All reanalyzed cell annotations were further validated by the marker gene expression reported in the original publication.

### Construction of the human embryonic reference

The human embryonic reference was established by integrating published datasets, included six sets of data spanning zygote early embryos, in vitro cultured blastocysts, 3D in vitro cultured human blastocysts up to pregastrulation stages and a CS7 human gastrula. Integration utilized the fastMNN from the batchelor (v.1.6.2) package. The top 4,000 variable genes were selected using the ‘SelectIntegrationFeatures’ function from the Seurat package. Four out of the six reference datasets were preimplantation datasets. To mitigate voting bias during the ‘SelectIntegrationFeatures’ function, the top 2,000 variable genes were first selected from the four preimplantation datasets. Subsequently, a combination consideration was applied to the remaining postimplantation dataset and CS7 dataset. The linear merge of batches followed the order of embryo developmental time points in the datasets. After obtaining the MNN-corrected PCA subspace results from the fastMNN calculation, the UMAP dimensional reduction was calculated using the ‘umap’ function of the uwot package (v.0.1.14) (https://CRAN.R-project.org/package=uwot), employing the top 50 MNN-corrected PCA subspace. The entire dataset was clustered using the Leiden algorithm (‘RunLeiden’ function from the Seurat package)^86^, utilizing the same neighborhood graph constructed from the corrected PCA subspace. In epiblasts belonging to clusters C1 and C10, 12 were identified as early and late epiblasts, respectively. Primitive endoderm cells were split as early (clusters C1 and C22) and late hypoblasts (cluster C11), respectively (Extended Data Fig. 1b–d). Throughout this process, the grand mean values (grand.centers) values, singular value decomposition results from MNN corrections, variation along the batch vectors, UMAP model and cell clustering were recorded for query dataset projection and identity prediction.

### Identification of marker genes and regulatory activity of transcription factors within human embryonic datasets

Lineage marker genes were identified using the ‘FindAllMarkers’ function with default parameters, with adjusted *P* value cutoff at 0.05. To identify the markers conserved in each lineage of the primate, marker detection was performed within each species and *P* values were combined using ‘stouffer’ function from poolr package (v.1.1-1)^87^ and followed by adjustment. A gene was considered conserved if its expression, with an adjusted *P* value of less than 0.05, was similar in all species. If the gene with an adjusted *P* value of less than 0.05 was highest in only one species’ lineage, but not similarly the highest in other two species corresponding lineage, it was considered a species-specific lineage marker gene. The categories hypoblast, DE and YSE are referred to as ‘Endoderm’, while preimplantation TE, CTB, STB and EVT are grouped as ‘TEs’. Additionally, ‘ExE_Mech’, ‘Stalk’ and ‘ExE_Mes’ are consolidated as the ‘ExE_Mes_stalk’ group, representing the extraembryonic lineage. Adv_Mes and axial mesosderm (Axial_Mes) from humans have been excluded from the mesoderm groups due to their similarity with extraembryonic lineages or distinction from the main mesoderm. ‘EmDisc’ from marmoset has been included in the epiblast group as the majority of them overlap with the epiblast. Pseudotime trajectory was computed using the R package slingshot (v.2.6.0)^27^, facilitating computation of lineage structures in a low-dimensional space. In summary, precomputed cell embeddings and annotations obtained from a human embryonic reference served as input for the ‘slingshot’ function. The start cluster was set to ‘zygote’, followed by the application of the ‘slingPseudotime’ function to infer individual pseudotime. The cells, categorized into prelineages (zygote, 2–4 cell, 8 cell, morula and E5 prelineage), ICM, epiblast, hypoblast, TE and CTB, were utilized to infer the main trajectories. The ‘DynamicHeatmap’ function from the R package SCP (v.0.5.6, available at https://github.com/zhanghao-njmu/SCP) was employed to identify transcriptional factor genes significantly associated with the pseudotime in the three main trajectories start from the morula stage. Batch-corrected gene expression from six human embryonic datasets was initially calculated using the ‘mnnCorrect’ function from the batchelor package. The resulting expression profiles were then utilized to identify regulatory modules by inferring coexpression with transcription factors through the ‘pyscenic grn--method grnboost2’ command. Each coexpression module served as input for *cis*-regulatory motif analyses conducted by running ‘pyscenic ctx’ with the following motif collections: ‘hg38__refseq-r80__10kb_up_and_down_tss.mc9nr.feather’, ‘hg38__refseq-r80__500bp_up_and_100bp_down_tss.mc9nr.feather’ and ‘motifs-v9-nr.hgnc-m0.001-o0.0.mod.tbl’^21^. Subsequently, the area under the curve values for each regulon were computed using ‘pyscenic aucell’. The top five valid regulons were further selected by requiring regulon activity in more than 1% of total cells and an average area under the curve value exceeding 0.05 for cells belonging to the same lineages.

### Cross-species integration

Integration of human datasets with cynomolgus and marmosets data included following steps. First, cynomolgus gene IDs were converted to human gene symbols using the Ensembl ortholog list^88^. Marmoset genes were merged based on shared gene names within the human dataset. Subsequently, rescaled normalization using ‘multiBatchNorm’ was performed for each species. Cells from Yang et al.^25^ were down sampled to 2,000. The top 3,500 variable genes for cynomolgus and marmosets were selected using ‘SelectIntegrationFeatures’ function, and cross-checked with the top variable genes used in the human embryo reference to confirm overlapping genes amongst all three species. From the 14,978 genes shared between the three species, the 6,005 top variable genes were identified. Of these, 544 or 1,597 genes were identified as the top variable genes in all three species or in any two species, respectively. Additionally, the top 500 species-unique variable genes were included, resulting in a final selection of 3,641 genes for the integration of all three species. To preserve the specificity of each individual species while emphasizing commonalities, the ‘mnnCorrect’ function from the batchelor package was used to calculate batch-corrected expression within each species separately. Batch-corrected expression of the three species, which removed within-species batch differences, served as input for the ‘FindIntegrationAnchors’ and ‘IntegrateData’ functions from the Seurat package for integration. Subsequently, the top 20 PCs were calculated using the ‘RunPCA’ function and utilized for UMAP dimensional reduction with the ‘RunUMAP’ function.

For data integration of preimplantation blastoids with in vitro cultured postimplantation cynomolgus monkey embryos (wild-type, day 14)^25^, normalization was performed separately for each dataset. Each human blastoid dataset and cynomolgus monkey dataset were integrated using the ‘RunfastMNN’ function (wrapped in the R SeuratWrappers package (v.0.3.0)) with 2,000 anchor features and the top 25 PCs. The resulting components were used for UMAP dimensional reduction with the ‘RunUMAP’ function.

### Projection of query dataset on human embryonic reference

To project the query dataset without influencing original dimensional reduction of reference dataset, the following steps were taken:

1. Normalization: when incorporating a query dataset, it is rescaled to the lowest coverage batch of the reference dataset^9^. The rescaling factor used for the reference dataset is also applied to the query dataset;
2. MNN correction and PCA projection: implementing MNN correction to remove the batch effect assumes that the presence of MNNs defines the most similar cells of the same type across batches and that the batch effect is almost orthogonal to the biological subspace. The sample size of the query dataset and the value of *K*, which defines the number of neighbors checked, can influence the prediction of MNN pairs. Therefore, we tested the accuracy of MNN pairs originating from the same reference lineages under different parameters (Extended Data Fig. 4a,b). Using the dataset from Ai et al.^43^, containing the largest number of embryonic cells, as our test dataset, we examined the *F*-score values of corrected MNN pairs under different values of *K* and downsampling sizes, as well as the processing time (Extended Data Fig. 4b). Accordingly, the query dataset was divided into several 200 subsamples and the default value of *K* was set to 30, except when the number of query samples was less than 50, in which case *K* was set to 5. In addition, each sample was randomly selected five times to form different subsamples to prevent bias introduced by downsampling. For the entirety of the MNN calling process, the ‘findMutualNN’ function from the batchelor R package was employed on cosine normalization values between the query dataset and each reference dataset. In our reference dataset, distinguishing between the two cell populations, TE and amnion, poses challenges due to their gene expression similarities. To further enhance accuracy, query cells assigned to both amnion and TE reference MNN pairs were discarded from downstream analysis to circumvent inaccurate calculation based on the cells with ambiguous signatures. Instead, only cells with clear signatures for either amnion or TE were retained, thus better distinguishing cell types. In addition, MNN pairs with low correlation coefficients and those belonging to reference lineages where the top 20 correlation coefficients were less than 0.5 were filtered out due to poor quality. Subsequently, the query cosine-normalized data underwent removal of the same grand mean values (grand.centers) of each gene from reference dataset construction. This was followed by a dot product calculation with left singular vectors from singular value decomposition in the reference construction and removing variation along the reference batch correction vector (orthogonalization) using the internal functions ‘.orthogonalize_other’ and ‘.center_along_batch_vector’, previously wrapped in the ‘fastMNN’ function, to place the query dataset into the same corrected batch-corrected PCA space as the reference datasets. As described in the original paper^18^, cell-specific batch correction vectors were calculated based on identified MNN pairs and a further batch correction on PCA space was performed using internal functions ‘.compute_correction_vectors’ and ‘.adjust_shift_variance’, previously wrapped in the ‘mnnCorrect’ function from batchelor (v.1.6.2)^18^;
3. UMAP calculation: after obtaining batch-corrected PCA subspace results for the query dataset, 2D UMAP projections were calculated using the ‘umap_transform’ function based on the previously mentioned reference UMAP model.

To filter out nonrelated cells, a Spearman correlation was calculated between the query data and the reference dataset using cosine normalization values. To set the threshold for filtering irrelevant cells, processed expression files from unrelated datasets, including five scRNA-seq datasets from human pancreas, macrophage, fetal kidney development and liver tissues, were downloaded from https://cblast.gao-lab.org/ to test the correlation coefficient with human embryonic reference cells^89–93^. In our analysis, cells with a mean top correlation coefficient (calculated within top 20 correlated reference cells) less than 0.5 were considered nonrelated. In addition, before the entire projection calculation, raw counts for cells from 10x datasets stratified by different time point or treatment with similar expression patterns were aggregated within neighborhood nodes, as calculated by miloR package (v.1.2.0)^81^. This aggregation enhances our MNN calculation and correlation calculation. ‘prop’ were set to 0.15 for ‘makeNhoods’ function from miloR package based on the performance testing results (Extended Data Fig. 4a,b).

### Prediction of cell identities

To predict cell identities, we first trained a SVM classifier for each lineage in latent space. Specifically, the embryonic reference dataset was split into training and testing data using fivefold cross-validation. Within each fold, we performed a grid search to find the best hyperparameters by tuning the *C* and gamma parameters of the radial basis function kernel (svmRadial). The optimal combination of hyperparameters was selected based on the ‘Kappa’ metric value. The R packages caret (v.6.0-88)^94^ and e1071 (v.1.7-13) (https://cran.r-project.org/web/packages/e1071/) were used here. For the query dataset, we then transformed the data into the same *N* dimensions as the reference using the ‘umap_transform’ function and was predicted utilizing the best-trained models for that dimension. Cells with the highest prediction probability no less than 0.5 were assigned to the corresponding reference lineages. Cells with the highest prediction probability lower than 0.5 were labeled as ‘ambiguous’. Additionally, based on the assumption that MNN pairs represent the most similar cells of the same type across batches, predicted lineages without support from MNN pairs were labeled as ‘ambiguous’, possibly due to inaccurate UMAP transformation or batch correction for query cells. For query datasets aggregated with neighborhood nodes, cells contributing to the neighborhood were assigned the same predicted annotation as that for the neighborhood. Cells with multiple predicted lineages were reported by the highest prediction probability. Cells that failed to form a neighborhood during miloR calculation were labeled as ‘nb_failed’.

To evaluate the dimensionality of the latent space that yields the best classifier performance, the original 50-dimensional PCA-corrected space of the reference dataset was also transformed into 2- (as used for visualization), 5-, 10- and 20-dimensional UMAP latent space. We evaluated the overall performance for each best model trained on *n* = 2, 5, 10 and 20 UMAP latent space or original *n* = 50 in PCA space. The given 11 embryonic datasets were transformed into the same *N* dimensions as the reference dataset using the ‘umap_transform’ function. The best models trained were then used to evaluate overall performance. Transforming into a 20-dimensional latent space yielded the best performance based on the kappa values of the predictions for all embryonic data (Extended Data Fig. 4c).

The R packages SingleR (v.1.4.1)^45^, scmap (v.1.12.0)^46^ and ScType (v.6db9eef)^47^ with default settings were used to compare the cell type annotations (Extended Data Fig. 4d). Gene expression and rescaled log-transformed normalization matrices of all six embryonic reference datasets were merged into one input reference dataset for SingleR, or served as a separate reference list for the ‘scmapCluster’ function from scmap. The top 15 identified lineage marker genes were used as the reference input for the ‘sctype_score’ function from ScType.

### Module score calculation

The predicted lineage scores for each cell were calculated using the ‘AddModuleScore’ function from Seurat package, incorporating the top 15 identified lineage marker genes.

### Detection of top DEGs among amnion, PriS and ExE_Mes cells and TE

The ‘FindMarkers’ function utilizing the two-sided ‘wilcox’ test from the Seurat package was employed for differential expression analysis.

Gene expression of amnion, PriS and extraembryonic cells from Tyser et al.^8^ were compared with TE cells from Petropoulos et al.^6^, Yanagida et al.^9^, Meistermann et al.^10^ and Xiang et al.^7^. The TE cells from Xiang et al.^7^ were categorized into preimplantation TE and postimplantation CTB, STB and EVT. All TE populations were compared with amnion, PriS or ExE_Mes cells. Since cells of late lineages and TE lineages were not from the same dataset, batch difference could influence DEG detection. For a gene to be considered truly differentially expressed, it had to meet four additional criteria: (1) average expression level in upregulated lineages greater than 10; (2) log_2_(fold change) greater than 0.25 in all five comparisons; (3) adjusted *P* value less than 0.05 in at least four comparisons; and (4) the percentage of cells expressing the gene in the highly expressed group greater than 50% and the percentage of cells expressing the gene in the lowly expressed group less than 25%. To ensure consistency, DEGs located on the sex chromosome were excluded from analysis, considering only the male embryo included in Tyser et al.^8^.

### Detection of DEGs between naive/primed-derived TLC and preimplantation embryonic TE

The gene expression profiles of predicted naïve and primed TLCs were compared with TE cells obtained from studies by Petropoulos et al.^6^, Yanagida et al.^9^, Meistermann et al.^10^ and Xiang et al.^7^, specifically focusing on preimplantation TE cells (E6 to E7). When comparing all TE populations, considering that the nonembryonic datasets were all generated on 10x platforms, for a gene to be considered truly differentially expressed, it needed to meet four additional criteria: (1) log_2_(fold change) >0.25 in at least three comparisons; (2) adjusted *P* value <0.05 in at least three comparisons; (3) adjusted merged *P* values combined by ‘stouffer’ function less than 0.05; and (4) the percentage of cells expressing the gene in the highly expressed group greater than 50% and percentage of cells expressing the gene in the lowly expressed group less than 25% in at least three comparisons.

### Detection of DEGs among blastoid-derived ELC, HLC and TLC versus corresponding preimplantation embryonic lineages

Gene expression profiles of ELC, HLC and TLC from studies by Yanagida et al.^9^, Kagawa et al.^48^ and Yu et al.^64^ were compared with preimplantation embryonic related lineage cells from studies including Petropoulos et al.^6^, Yanagida et al.^9^, Meistermann et al.^10^ and Xiang et al.^7^, respectively. When comparing blastoid lineage-like cells with embryonic reference cells, given the limited number of reference cells for the preimplantation hypoblast in studies by Yanagida et al.^9^, Meistermann et al.^10^ and Xiang et al.^7^ (14, 2 and 7 cells, respectively), hypoblast cells from Meistermann et al.^10^ were excluded in the reference comparison. Comparisons between HLC and primitive endoderm cells from Yanagida et al.^9^ and Xiang et al.^7^ *P* values were considered significant. To be considered truly differentially expressed, a gene had to meet three additional criteria, including: (1) log_2_(fold change) >0.25 in at least three comparisons; (2) having an adjusted *P* value <0.05 in at least three comparisons; and (3) adjusted merged *P* values combined by ‘stouffer’ function <0.05. GSEA was performed using the ‘GSEA’ function from the clusterProfiler package (v.3.18.1)^95^, with the average log_2_(fold change) from four comparisons with embryonic lineages as input. WikiPathway annotations and gene sets for GSEA were downloaded from the Molecular Signatures Database^68,96^. Significantly regulated WikiPathways were identified as those with a Benjamini–Hochberg-adjusted *P* value less than 0.05.

### Extension of human embryonic reference

Two additional datasets were included: spatial transcriptomics from a CS8 human embryo^60^ and 10x-sequenced single-cell transcriptomes of STB, EVT and villous CTB from first-trimester placentas^61^. These were added to the original six reference datasets to test the extension capabilities of our reference construction strategy. Since the sequencing depth of the spatial transcriptomics and 10x single-cell transcriptomes were 130 and 20 times lower, respectively, compared with the six non-droplet-based datasets, we aggregated the raw gene expression of the CS8 spatial transcriptomics^60^ two times using the same strategy for sparse query datasets by miloR. Similarly, for the 10x-sequenced dataset, one-time aggregation was employed for the first-trimester placentas cells. After aggregation, reference construction followed the same normalization, MNN correction and UMAP reduction steps as for our original reference construction, utilizing the top 4,500 variable genes and 50 PCs. Query datasets, including TLCs derived from naive and primed hPS cells and organoids derived from the first trimester^59^, were projected onto this extended reference following the same processing steps as for the original reference.

### Statistics and reproducibility

On the basis of the prediction performance, the downsampling sample size in Fig. 2a was determined as 200. No data were excluded from the analyses. The samples were not randomized unless specified in Extended Data Figs. 3a,d and 8a,b,d. Cells from Yang et al.^25^ were downsampled to 2,000 in Extended Data Fig. 3a. Cells more than 200 cells were downsampled to 200 in Extended Data Figs. 3d and 8a,b,d.

### Web interface

A shiny app generated by shinyCell^79^, which includes the above integration, as our early embryogenesis prediction tool can be browsed at http://petropoulos-lanner-labs.clintec.ki.se.

### Reporting summary

Further information on research design is available in the Nature Portfolio Reporting Summary linked to this article.

## Online content

Any methods, additional references, Nature Portfolio reporting summaries, source data, extended data, supplementary information, acknowledgements, peer review information; details of author contributions and competing interests; and statements of data and code availability are available at 10.1038/s41592-024-02493-2.

## Supplementary information

Supplementary Information Supplementary Figs. 1–8.

Reporting Summary

Supplementary Data 1. UMAP coordinates of human and cross-species integration, including cell annotations and corresponding publications.

Supplementary Data 2. Transcription factors associated with epiblast, hypoblast and TE trajectories. *P* values were calculated by a two-sided test and adjusted using the ‘Holm’ method.

Supplementary Data 3. Lineage markers genes identified in human early embryos. *P* values were calculated by a two-sided ‘Wilcox’ test and adjusted using the ‘Bonferroni’ method.

Supplementary Data 4. Conserved or species-specific lineage markers genes identified in human, cynomolgus monkey and marmoset. *P* values were calculated by a two-sided ‘Wilcox’ test, combined using the ‘stouffer’ function and then adjusted using the ‘Bonferroni’ method.

Supplementary Data 5. DEGs identified between naive and primed cells and between preimplantation and late lineages of embryonic TE cells. *P* values were calculated by a two-sided ‘Wilcox’ test and adjusted using the ‘Bonferroni’ method.

Supplementary Data 6. DEGs identified comparing naive and primed-derived TLC with and preimplantation embryonic TE cells. *P* values were calculated by a two-sided ‘Wilcox’ test and adjusted using the ‘Bonferroni’ method.

Supplementary Data 7. DEGs identified between early and late epiblast, early and late hypoblast, and among TE, CTB, EVT and STB lineages. *P* values were calcuated by two-sided ‘Wilcox’ test, and combined by the ‘stouffer’ function then adjusted by the ‘Bonferroni’ method.

Supplementary Data 8. DEGs identified and GSEA results comparing blastoids with and preimplantation embryonic cells. *P* values were calcuated by two-sided ‘wilcox’ test, and combined by the ‘stouffer’ function then adjusted by the ‘Bonferroni’ method.

Supplementary Data 9. Datasets used in this analysis.

## Source data

Source Data Fig. 1. Statistical source data.

Source Data Fig. 2. Statistical source data and download link for prediction results.

Source Data Fig. 3. Statistical source data and download link for prediction results.

Source Data Fig. 4. Download link for prediction results.

Source Data Fig. 5. Download link for prediction results.

Source Data Fig. 6. Statistical source data.

Source Data Extended Data Fig. 1. Statistical source data.

Source Data Extended Data Fig. 2. Statistical source data.

Source Data Extended Data Fig. 3. Statistical source data.

Source Data Extended Data Fig. 4. Statistical source data.

Source Data Extended Data Fig. 6. Statistical source data.

Source Data Extended Data Fig. 7. Statistical source data.

Source Data Extended Data Fig. 8. Statistical source data.

## Data Availability

Datasets utilized in this study were obtained as summarized in Supplementary Data 9. These include 13 human embryonic datasets covering various stages of embryogenesis (Yan et al.^11^ (GSE36552), Xiang et al.^7^ (GSE136447), Tyser et al.^8^ (E-MTAB-9388), Yanagida et al.^9^ (GSE171820), Meistermann et al.^10^ (PRJEB30442), Zhou et al.^44^ (GSE109555), Xue et al.^41^ (GSE44183), Molè et al.^12^ (E-MTAB-8060), Ai et al.^43^ (PRJCA017779), Blakeley et al.^42^ (GSE66507), Petropoulos et al.^6^ (E-MTAB-3929), Xiao et al.^60^ (HRA005567) and Vento-Tormo et al.^61^ (E-MTAB-6701)). Additionally, we included seven preimplantation blastoid models (Yu et al.^64^ (GSE150578), Liu et al.^65^ (GSE156596), Kagawa et al.^48^ (GSE177689), Fan et al.^66^ (GSE158971), Sozen et al.^67^ (GSE178326), Yu et al.^16^ (GSE210962) and Yanagida et al.^9^ (GSE171820)); seven post-implantation stem cell-based models (Karvas et al.^76^ (GSE226794), Hislop et al.^75^ (GSE247111), Weatherbee et al.^13^ (GSE218314), Oldak et al.^74^ (GSE239932), Pedroza et al.^72^ (GSE208195), Liu et al.^73^ (GSE232861) and Ai et al.^43^ (PRJCA017779)); three studies involving naive cells giving rise to TLCs (Io et al.^57^ (GSE167924), Guo et al.^50^ (GSE166422) and Osnato et al.^54^ (E-MTAB-10018)); three studies using primed human embryonic cells giving rise to TLCs (one generated in house (GSE254641), Soncin et al.^55^ (GSE182791) and Ohgushi et al.^56^ (GSE196365)); two studies analyzing 8CLCs (Mazid et al.^62^ (CNP0001454) and Yoshihara et al.^63^ (E-MTAB-10581)); one study with a PASE model (Zheng et al.^17^ (GSE134571)); and one study from human trophoblast organoids (Shannon et al.^59^ (GSE216244)). Furthermore, we included two embryonic datasets from *Callithrix jacchus* (marmoset) (Bergmann et al.^31^ (E-MTAB-9367) and Boroviak et al.^28^ (E-MTAB-7078)) and three embryonic datasets from *Macaca fascicularis* (crab-eating macaque) (Nakamura et al.^29^ (GSE74767), Ma et al.^30^ (GSE130114) and Yang et al.^25^ (GSE148683)). Yanagida et al.^9^ 2021 and Ai et al.^43^ both contain embryonic and stem cell model data. The processed dataset, including predicted annotations, UMAP and sorted cell counts, can be retrieved from https://petropoulos-lanner-labs.clintec.ki.se/dataset.download.html. Source data are provided with this paper.

## Code availability

All original code has been deposited and is available via Zenodo at https://zenodo.org/records/12189592 (ref. ^97^) with a GPL-3.0 license for all versions. Any additional information required to reanalyze the data reported in this work paper is available from the lead contact upon request.
