# Author Correction: A comprehensive human embryo reference tool using single-cell RNA-sequencing data

**DOI:** 10.1038/s41592-025-02601-w

**Published:** 2025-01-24

**Authors:** Cheng Zhao, Alvaro Plaza Reyes, John Paul Schell, Jere Weltner, Nicolás M. Ortega, Yi Zheng, Åsa K. Björklund, Laura Baqué-Vidal, Joonas Sokka, Ras Trokovic, Brian Cox, Janet Rossant, Jianping Fu, Sophie Petropoulos, Fredrik Lanner

**Affiliations:** 1 Department of Clinical Science, Intervention and Technology, Karolinska Institutet, and Division of Obstetrics and Gynecology, Karolinska Universitetssjukhuset, Stockholm, Sweden; 2 Department of Integrative Pathophysiology and Therapy, Andalusian Molecular Biology and Regenerative Medicine Centre (CABIMER), Seville, Spain; 3 Stem Cells and Metabolism Research Program, University of Helsinki, Helsinki, Finland; 4 Folkhälsan Research Center, Helsinki, Finland; 5 Department of Mechanical Engineering, University of Michigan, Ann Arbor, MI USA; 6 Department of Biomedical and Chemical Engineering, Syracuse University, Syracuse, NY USA; 7 Department of Cell and Molecular Biology, National Bioinformatics Infrastructure Sweden, Science for Life Laboratory, Uppsala University, Uppsala, Sweden; 8 Department of Physiology, Faculty of Medicine, University of Toronto, Toronto, Ontario Canada; 9 Program in Developmental and Stem Cell Biology, Hospital for Sick Children, Toronto, Ontario Canada; 10 Department of Cell and Developmental Biology, University of Michigan Medical School, Ann Arbor, MI USA; 11 Department of Biomedical Engineering, University of Michigan, Ann Arbor, MI USA; 12 Département de Médecine, Université de Montréal, Montreal, Quebec Canada; 13 Centre de Recherche du Centre Hospitalier de l’Université de Montréal, Axe Immunopathologie, Montreal, Quebec Canada; 14 Ming Wai Lau Center for Reparative Medicine, Stockholm Node, Karolinska Institutet, Stockholm, Sweden

**Keywords:** Embryogenesis, Classification and taxonomy, Embryonic stem cells

Correction to: *Nature Methods* 10.1038/s41592-024-02493-2, published online 14 November 2024.

In the version of this article initially published, the surname of Ras Trokovic was misspelled (as Torokovic). In Extended Data Fig. 10c, the “+” entry in the DE row under “Karvas et al.” appeared as a minus sign. The corrections have been made in the HTML and PDF versions of the article.

