# Supplementary material for: A comprehensive human embryo reference tool using single-cell RNA-sequencing data
Source: Nat Methods. 2024 Nov 14;22(1):193–206. doi: 10.1038/s41592-024-02493-2 (PMC11725501; doi:10.1038/s41592-024-02493-2)
Supplement: Supplementary file 1 — Supplementary Figs. 1–8. [file 41592_2024_2493_MOESM1_ESM.pdf]

nature methods

Supplementary information https://doi.org/10.1038/s41592-024-02493-2

A comprehensive human embryo reference tool using single-cell RNA-sequencing data

In the format provided by the authors and unedited

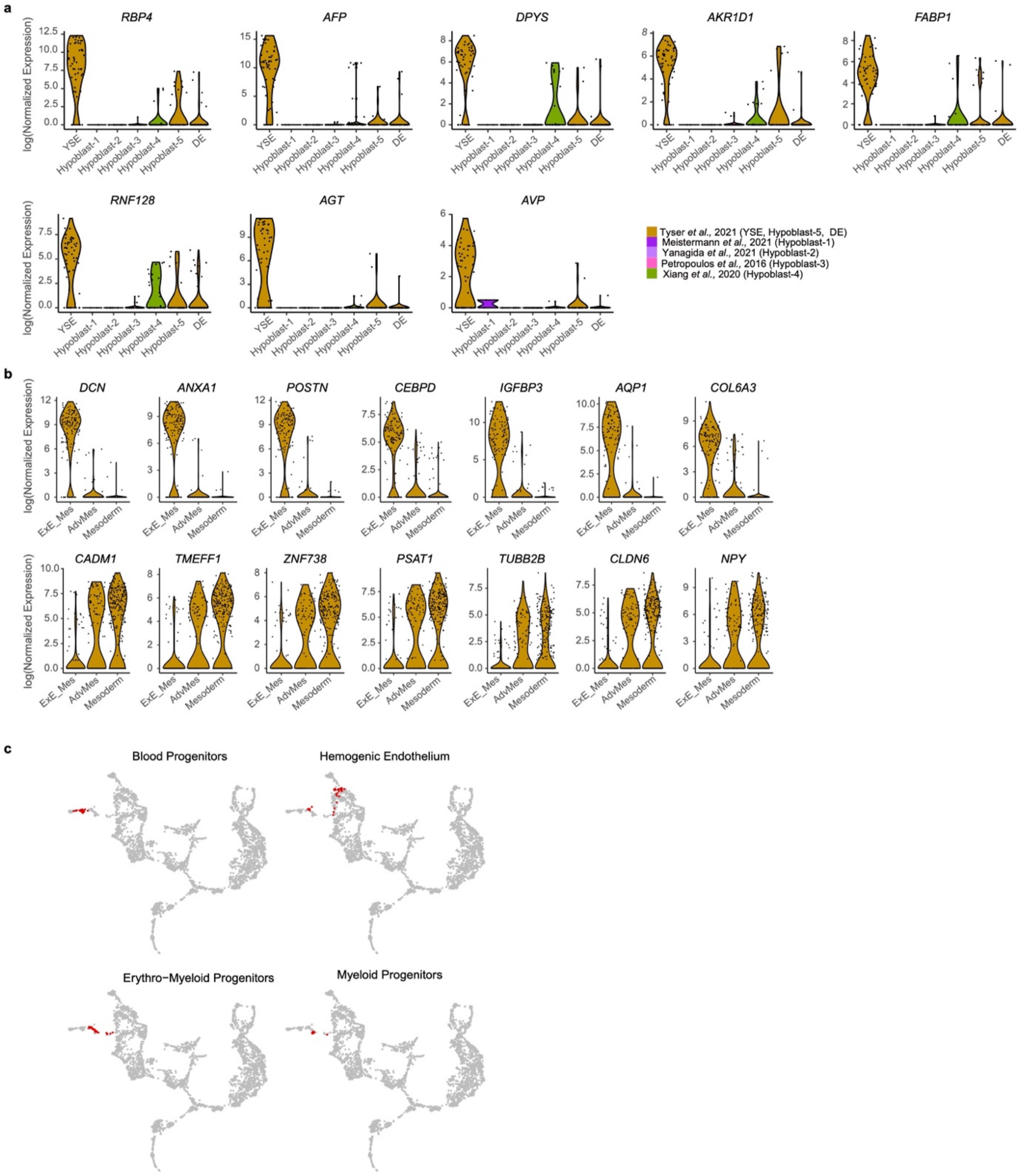

Supplementary Fig. 1 | Violin plot showing distinct markers expressed in YSE in comparison to hypoblast and DE (**a**), and top DEGs between ExE_Mes and embryonic mesoderm cells (**b**). **c**, Highlight cells belonging to each HEP subtype according to Tyser et al., 2021 annotations on reference UMAP.

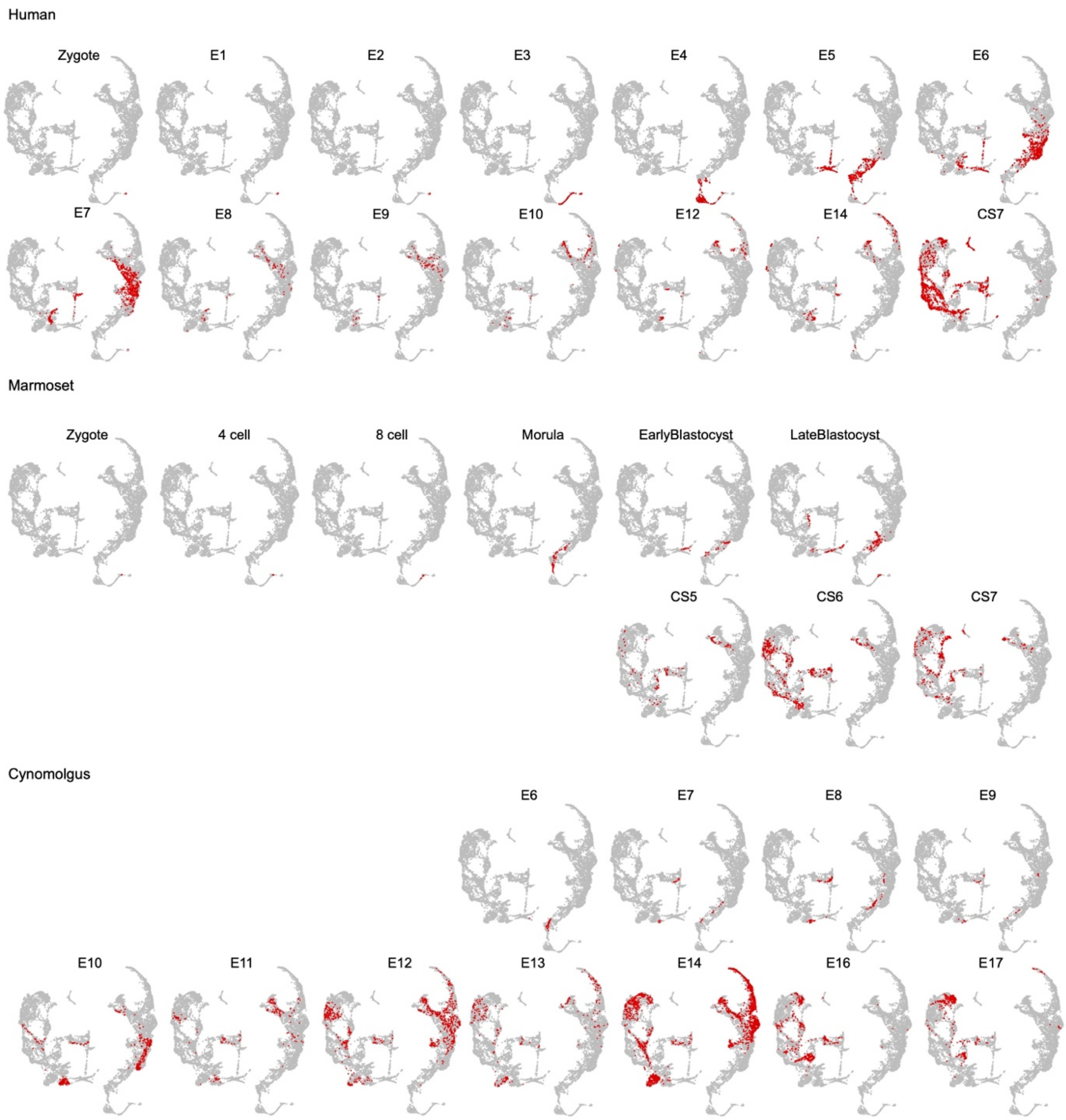

Supplementary Fig. 2 | Primate cross-species integration of embryonic datasets with cells highlighted from the different embryonic time points for each species.

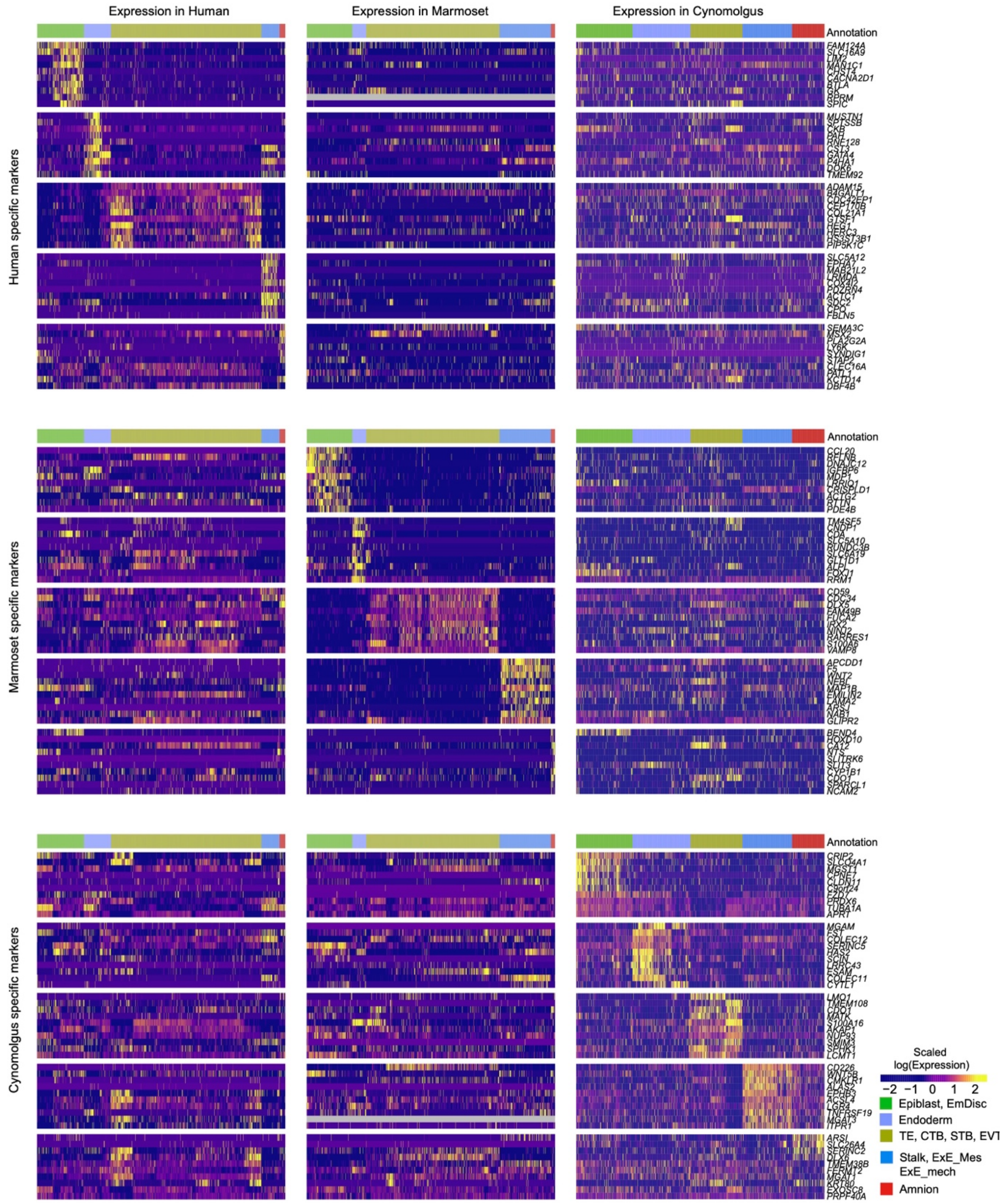

Supplementary Fig. 3 | Expression of the top 10 species-specific lineage marker genes in primate.

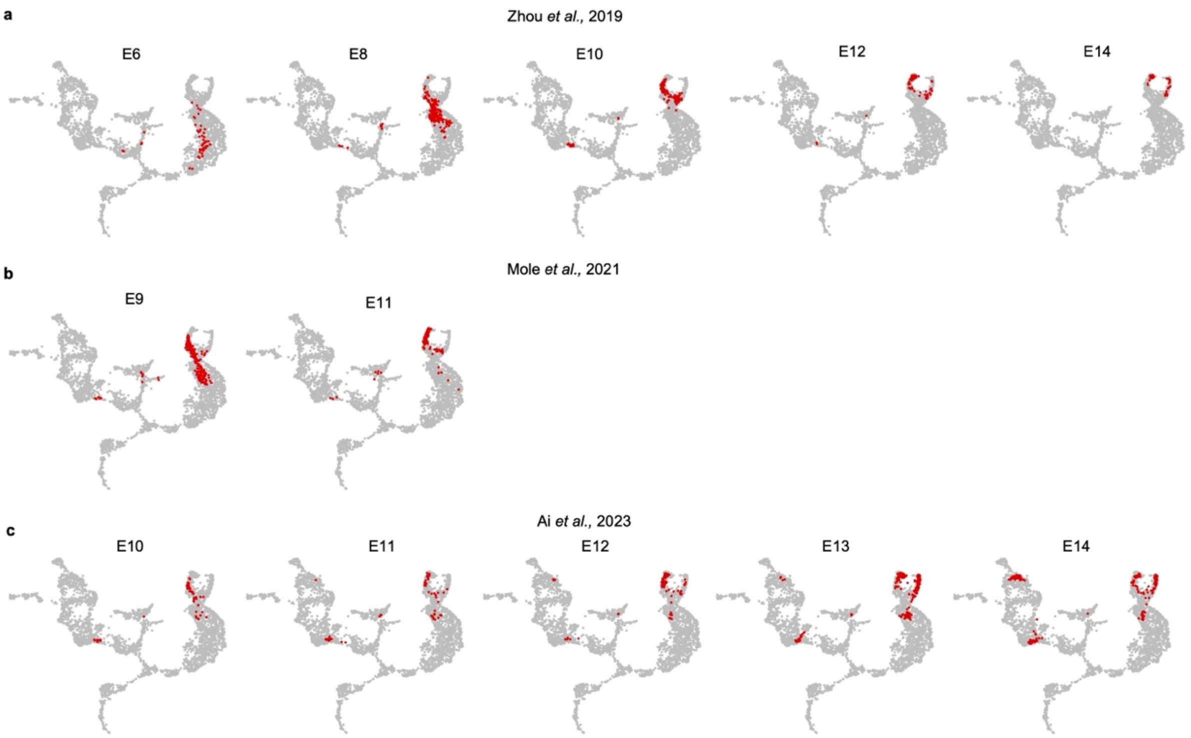

Supplementary Fig. 4

Highlighted cells (neighbourhood nodes) from different embryonic time points for Zhou et al., 2019 (**a**), Mole et al., 2021 (**b**) and Ai et al., 2023 (**c**) from the projection on embryonic reference.

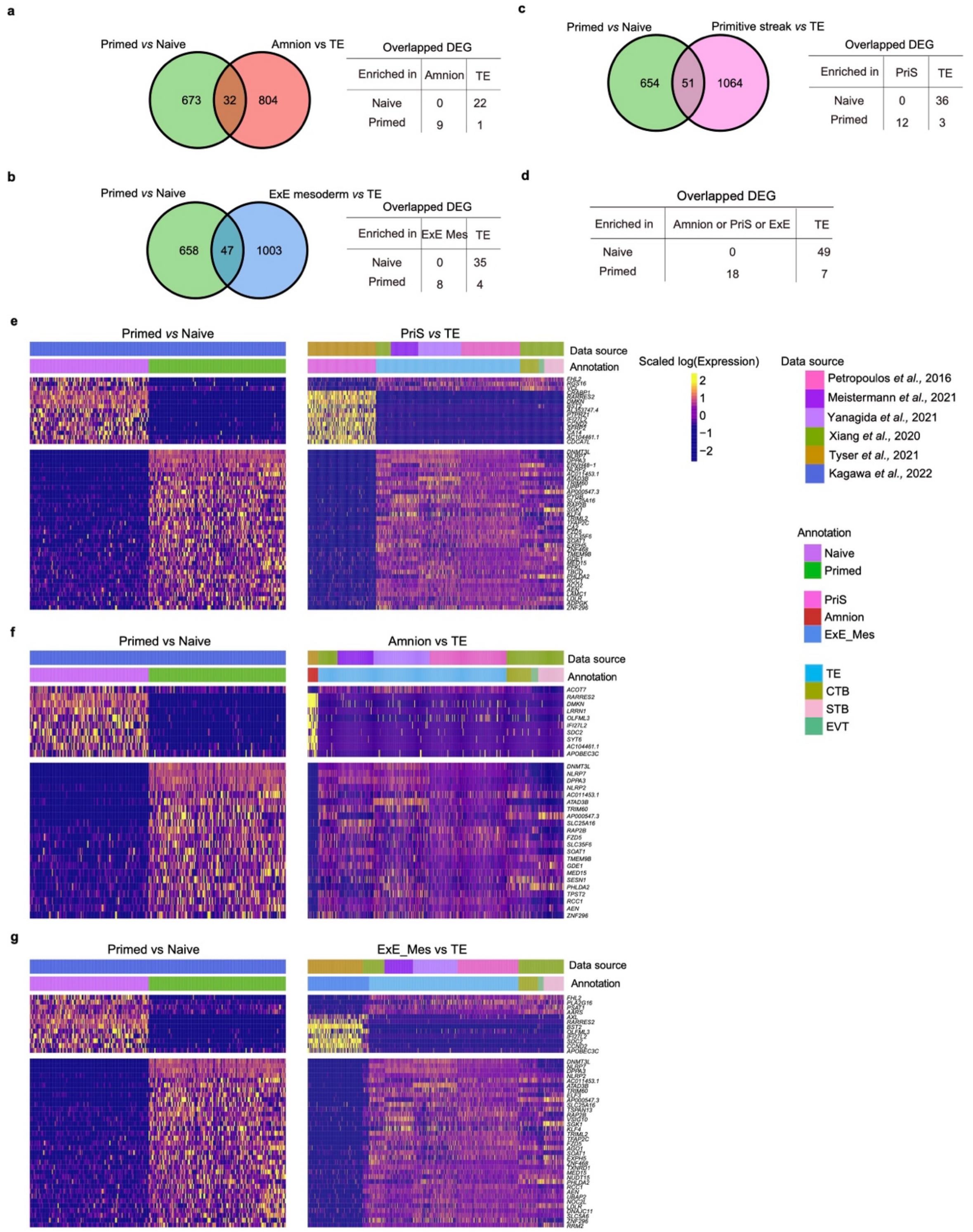

Supplementary Fig. 5

a, Venn diagram showing overlaps between naïve vs. primed DEGs and amnion vs. TE DEGs. The detailed number of DEGs with direction is included in the tables on the right. (**b**) ExE_Mes vs. TE DEGs, and (**c**) PriS vs. TE DEGs. **d**, The number of overlapping DEGs between late lineages vs. TE and naïve vs. primed. **e**, Heatmap showing expression of DEGs identified in naïve vs. primed and PriS vs. TE, (**f**) DEGs shared by naïve vs. primed and amnion vs. TE, and (**g**) DEGs shared by naïve vs. primed and ExE_Mes vs. TE.

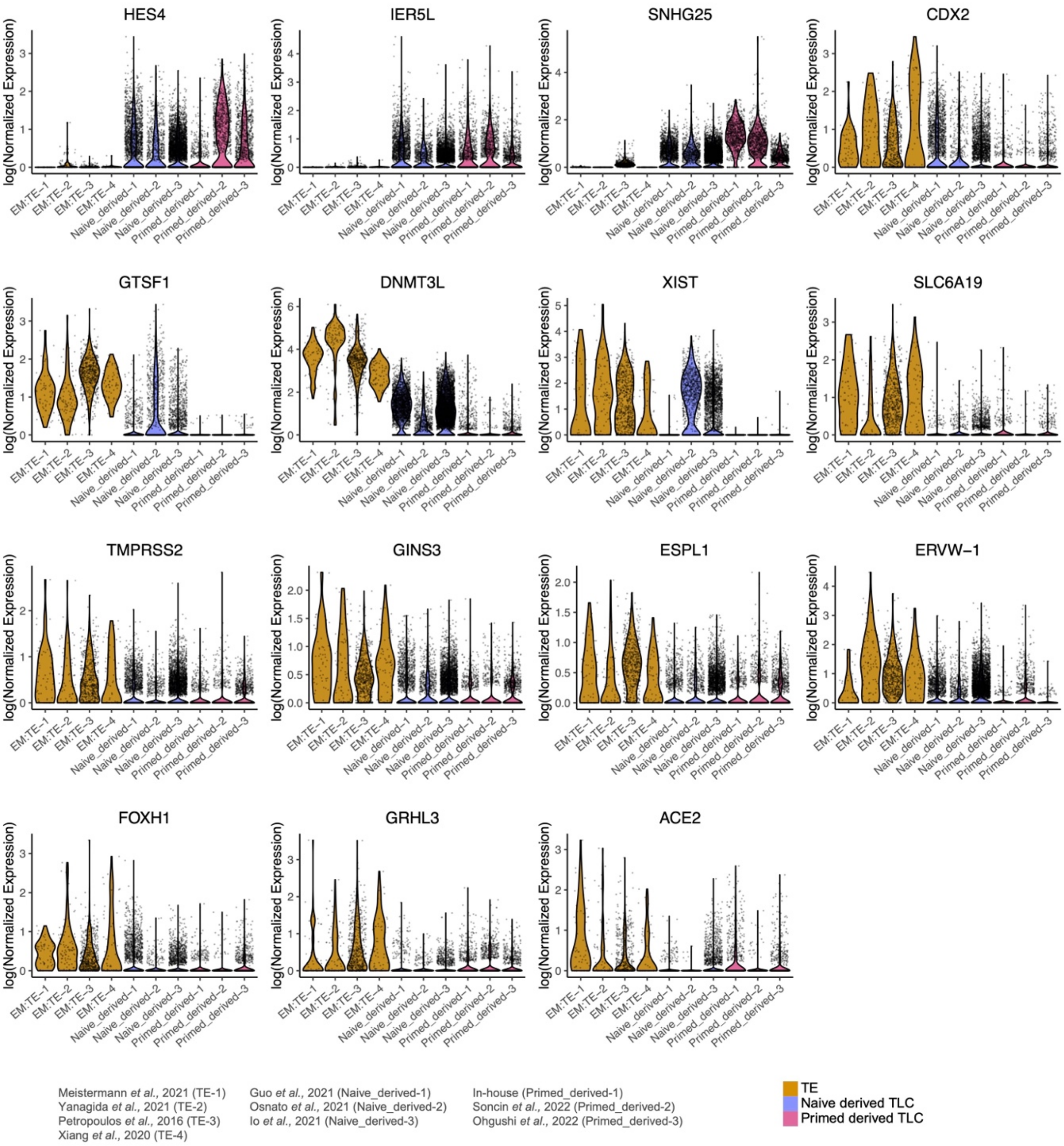

Supplementary Fig. 6 | Violin plot showing representative DEGs between naïve or primed derived TLCs and embryonic pr-eimplantation TE cells.

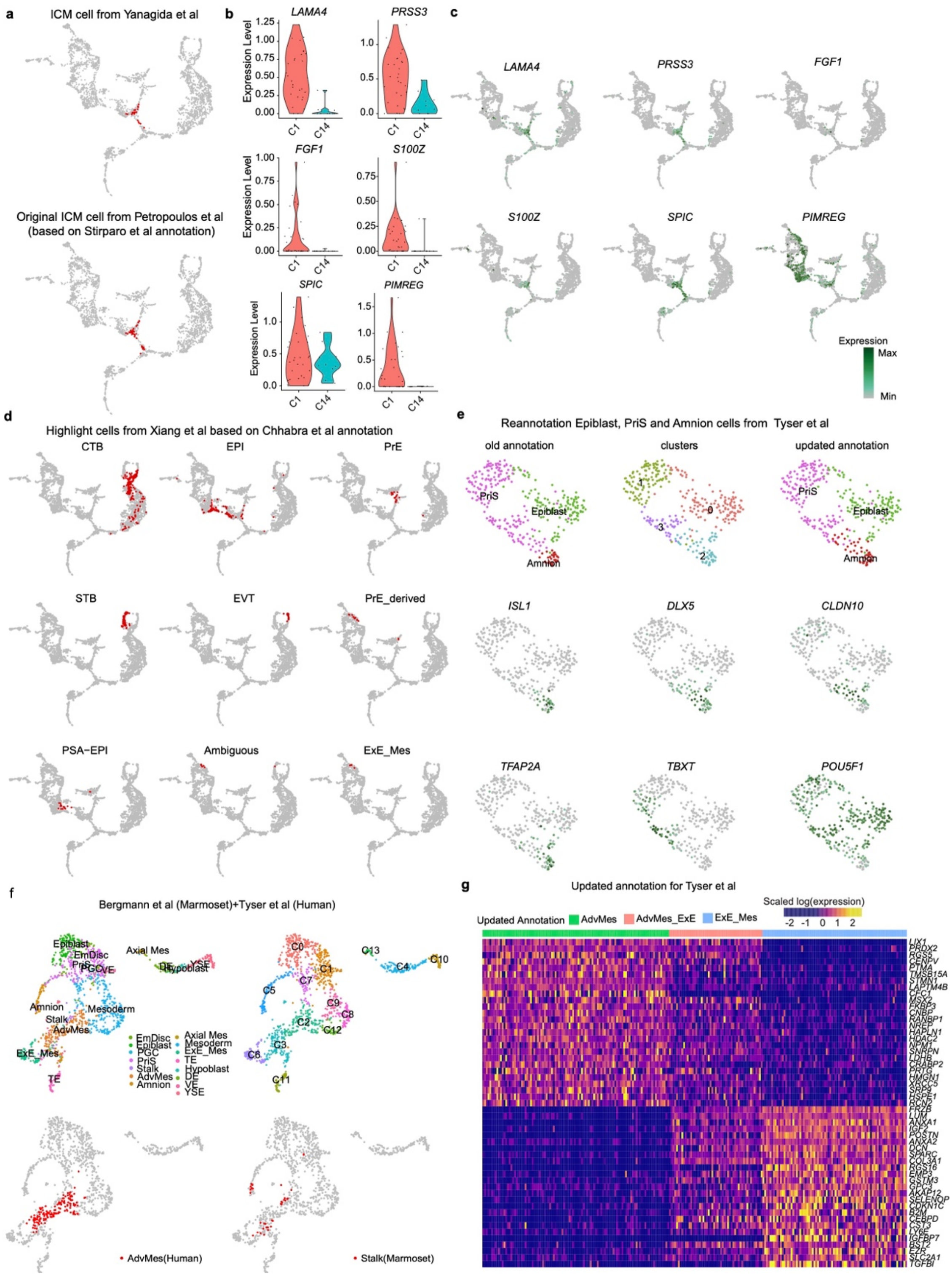

Supplementary Fig. 7 | Updating human embryonic reference annotation

a, Highlighted ICM cells from Yanagida et al., 2021 and Petropoulos et al., 2016 (based on the annotation from Stirparo et al.,2018). **b**, Violin plot showing the expression of ICM-specific markers in ICM cells from Petropoulos et al., 2016 based on the annotation from Stirparo et al., 2018. Cells were grouped by the unassigned cluster in the integration of six embryonic datasets. **c**, UMAP plot of cells showing the expression of ICM-specific markers. **d**. Highlighted cells from Xiang et al., 2020 based on the annotation from Chhabra et al., 2021. **e**. Reclustering of epiblast, primitive streak, and amnion cells from Tyser et al., 2021. At the top, cells are coloured

by the original annotation from Tyser et al., 2021, the unassigned cluster during reclustering, and the updated annotation. The expression of marker genes for amniotic ectoderm, primitive streak, and epiblast is shown at the bottom. **f**. UMAP projection of mutual nearest neighbours cross-species integration, including CS7 cells from humans (Tyser et al., 2021) and the marmoset embryo (Bergmann et al., 2022). The stalk cells from marmoset and the previously advanced mesoderm cells in humans are highlighted separately. **g**. Heatmap showing DEGs between advanced mesoderm cells (not belonging to cluster C3) and human extraembryonic mesoderm cells in human advanced mesoderm cells, reannotated extraembryonic mesoderm cells (AdvMes_ExE), and human embryo extraembryonic mesoderm cells.

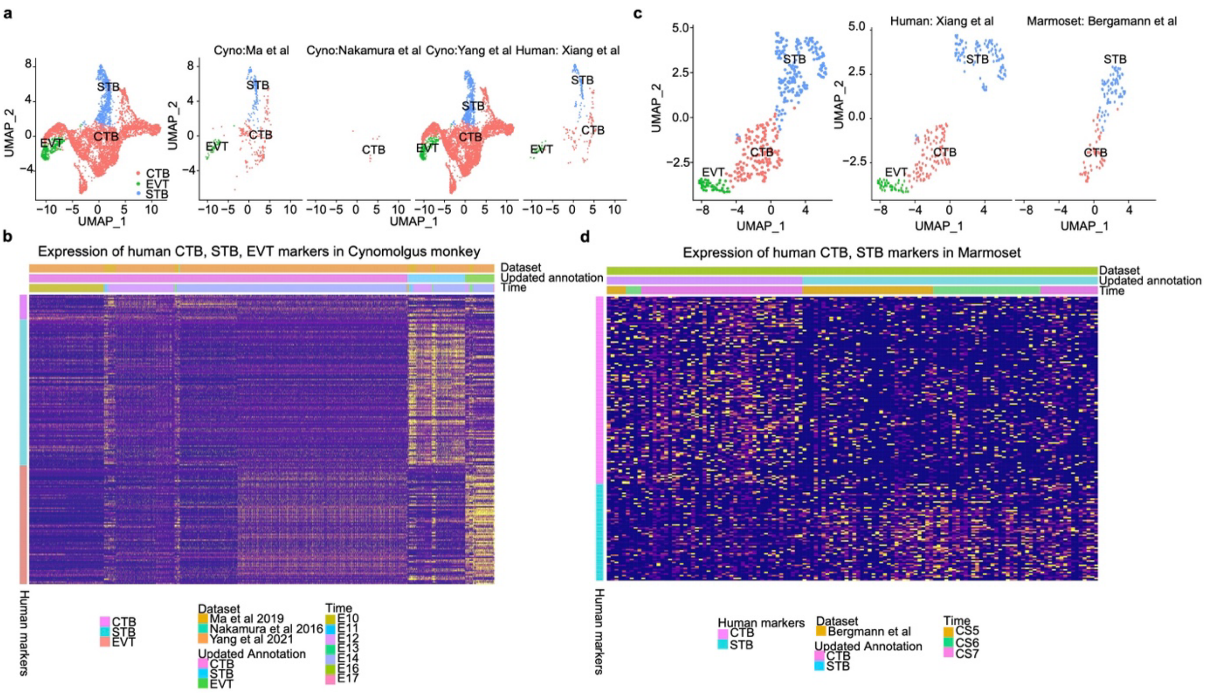

Supplementary Fig. 8

a, UMAP projection of Mutual Nearest Neighbours (MNN) cross-species integration, including human and cynomolgus monkey post-implantation TE cells. **b**, Heatmap showing the markers for human CTB, STB, and EVT which were also differentially expressed in identified cynomolgus monkey CTB, STB, and EVT cells. **c**, UMAP projection of Mutual Nearest Neighbours (MNN) cross-species integration, including human and marmoset post-implantation TE cells. **d**, Heatmap showing the markers for human CTB, STB, and EVT which were also differentially expressed in identified marmoset CTB, STB, and EVT cells.
