# Supplementary material for: A comprehensive human embryo reference tool using single-cell RNA-sequencing data
Source: Nat Methods. 2024 Nov 14;22(1):193–206. doi: 10.1038/s41592-024-02493-2 (PMC11725501; doi:10.1038/s41592-024-02493-2)
Supplement: Supplementary file 2 — Reporting Summary [file 41592_2024_2493_MOESM2_ESM.pdf]

nature portfolio

Corresponding author(s): Fredrik Lanner, Sophie Petropoulos

Last updated by author(s): Aug 4, 2024

Reporting Summary

Nature Portfolio wishes to improve the reproducibility of the work that we publish. This form provides structure for consistency and transparency in reporting. For further information on Nature Portfolio policies, see our Editorial Policies and the Editorial Policy Checklist.

Statistics

For all statistical analyses, confirm that the following items are present in the figure legend, table legend, main text, or Methods section.

n/a	Confirmed	
☐	☒	The exact sample size (*n*) for each experimental group/condition, given as a discrete number and unit of measurement
☐	☒	A statement on whether measurements were taken from distinct samples or whether the same sample was measured repeatedly
☐	☒	The statistical test(s) used AND whether they are one- or two-sided<br>*Only common tests should be described solely by name; describe more complex techniques in the Methods section.*
☒	☐	A description of all covariates tested
☐	☒	A description of any assumptions or corrections, such as tests of normality and adjustment for multiple comparisons
☐	☒	A full description of the statistical parameters including central tendency (e.g. means) or other basic estimates (e.g. regression coefficient) AND variation (e.g. standard deviation) or associated estimates of uncertainty (e.g. confidence intervals)
☐	☒	For null hypothesis testing, the test statistic (e.g. *F*, *t*, *r*) with confidence intervals, effect sizes, degrees of freedom and *P* value noted<br>*Give P values as exact values whenever suitable.*
☒	☐	For Bayesian analysis, information on the choice of priors and Markov chain Monte Carlo settings
☒	☐	For hierarchical and complex designs, identification of the appropriate level for tests and full reporting of outcomes
☐	☒	Estimates of effect sizes (e.g. Cohen's *d*, Pearson's *r*), indicating how they were calculated

Our web collection on statistics for biologists contains articles on many of the points above.

Software and code

Policy information about availability of computer code

Data collection	no software except "wget" command was used for data collection
Data analysis	The following software was used in this analysis:<br><br>Cellranger (v3.0.0 and v6.1.1) was used to process 10X scRNA-seq data.<br>STAR aligner (v2.5.3b) and RSEM (v1.3.0) were employed to map Smart-seq2 scRNA-seq reads.<br>Drop-seq tools (v2.5.1) were used to reprocess data from Ma et al., 2019 (PMID: 31672918).<br>The Seurat R package (v4.2.0) was utilized for scRNA-seq analysis.<br>The Leiden algorithm was applied to identify cell clusters for the human embryonic reference.<br>The scran R package (v1.14.6) was used for normalization.<br>The batchelor R package (v1.2.4) was employed for normalization and integration.<br>The uwot R package (v0.1.14) (https://CRAN.R-project.org/package=uwot) was used to compute the UMAP model.<br>The slingshot R package (v2.6.0) and the SCP R package (v0.5.6, available at https://github.com/zhanghao-njmu/SCP) were used to infer trajectories and identify trajectory-related genes.<br>The poolr R package (v1.1-1) was utilized to merge p-values.<br>The SeuratWrappers R package (v0.3.0) was used for data integration.<br>The miloR R package (v1.2.0) was used to calculate neighborhoods.<br>The caret (v6.0-88) and e1071 (v1.7-13) R packages were used to train SVM models and make predictions.<br>SingleR (v1.4.1), scmap (v1.12.0), and ScType (v6db9eef) were used to compare the prediction performance<br>The ShinyCell R package (v2.1.0) was used to build the Shiny website.<br>The clusterProfiler R package (v3.18.1) was employed to perform gene set enrichment analysis.

Early Embryogenesis Prediction Tool can be browsed at http://petropoulos-lanner-labs.clintec.ki.se

Custom code is available at https://zenodo.org/records/12189592.

For manuscripts utilizing custom algorithms or software that are central to the research but not yet described in published literature, software must be made available to editors and reviewers. We strongly encourage code deposition in a community repository (e.g. GitHub). See the Nature Portfolio guidelines for submitting code & software for further information.

Data

Policy information about availability of data

All manuscripts must include a data availability statement. This statement should provide the following information, where applicable:

- Accession codes, unique identifiers, or web links for publicly available datasets
- A description of any restrictions on data availability
- For clinical datasets or third party data, please ensure that the statement adheres to our policy

Datasets utilised in this study were obtained as summarised in Supplementary Data 9. These include thirteen human embryonic datasets covering various stages of embryogenesis (Yan et al. 2013(GSE36552, https://www.ncbi.nlm.nih.gov/geo/query/acc.cgi?acc=GSE36552); Xiang et al. 2020(GSE136447, https://www.ncbi.nlm.nih.gov/geo/query/acc.cgi?acc=GSE136447); Tyser et al. 2021(E-MTAB-9388, https://www.ebi.ac.uk/arrayexpress/experiments/E-MTAB-9388); Yanagida et al. 2021(GSE171820, https://www.ncbi.nlm.nih.gov/geo/query/acc.cgi?acc=GSE171820); Meistermann et al. 2021(PRJEB30442, https://www.ebi.ac.uk/ena/browser/view/PRJEB30442); Zhou et al. 2019(GSE109555, https://www.ncbi.nlm.nih.gov/geo/query/acc.cgi?acc=GSE109555); Xue et al. 2013(GSE44183, https://www.ncbi.nlm.nih.gov/geo/query/acc.cgi?acc=GSE44183); Molè et al. 2021(E-MTAB-8060, https://www.ebi.ac.uk/arrayexpress/experiments/E-MTAB-8060); Ai et al. 2023(PRJCA017779, https://ngdc.cncb.ac.cn/bioproject/browse/PRJCA017779); Blakeley et al. 2015(GSE66507, https://www.ncbi.nlm.nih.gov/geo/query/acc.cgi?acc=GSE66507); Petropoulos et al. 2016(E-MTAB-3929, https://www.ebi.ac.uk/arrayexpress/experiments/E-MTAB-3929); Xiao et al. 2024(HRA005567, https://ngdc.cncb.ac.cn/gsa-human/browse/HRA005567); Vento-Tormo et al. 2018(E-MTAB-6701, https://www.ebi.ac.uk/arrayexpress/experiments/E-MTAB-6701)). Additionally, we included seven preimplantation blastoid models(Yu et al. 2021 (GSE150578, https://www.ncbi.nlm.nih.gov/geo/query/acc.cgi?acc=GSE150578); Liu et al. 2021(GSE156596, https://www.ncbi.nlm.nih.gov/geo/query/acc.cgi?acc=GSE156596); Kagawa et al. 2022(GSE177689, https://www.ncbi.nlm.nih.gov/geo/query/acc.cgi?acc=GSE177689); Fan et al. 2021(GSE158971, https://www.ncbi.nlm.nih.gov/geo/query/acc.cgi?acc=GSE158971); Sozen et al. 2021(GSE178326, https://www.ncbi.nlm.nih.gov/geo/query/acc.cgi?acc=GSE178326); Yu et al. 2023(GSE210962, https://www.ncbi.nlm.nih.gov/geo/query/acc.cgi?acc=GSE210962); Yanagida et al. 2021(GSE171820, https://www.ncbi.nlm.nih.gov/geo/query/acc.cgi?acc=GSE171820)), seven post-implantation stem cell based models(Karvas et al. 2023(GSE226794, https://www.ncbi.nlm.nih.gov/geo/query/acc.cgi?acc=GSE226794); Hislop et al. 2024(GSE247111, https://www.ncbi.nlm.nih.gov/geo/query/acc.cgi?acc=GSE247111); Weatherbee et al. 2023(GSE218314, https://www.ncbi.nlm.nih.gov/geo/query/acc.cgi?acc=GSE218314); Oldak et al. 2023(GSE239932, https://www.ncbi.nlm.nih.gov/geo/query/acc.cgi?acc=GSE239932); Pedroza et al. 2023(GSE208195, https://www.ncbi.nlm.nih.gov/geo/query/acc.cgi?acc=GSE208195); Liu et al. 2023(GSE232861, https://www.ncbi.nlm.nih.gov/geo/query/acc.cgi?acc=GSE232861); Ai et al. 2023(PRJCA017779, https://ngdc.cncb.ac.cn/bioproject/browse/PRJCA017779)), three studies involving naïve cells giving rise to trophectoderm (TE)-like cells(Io et al. 2021(GSE167924, https://www.ncbi.nlm.nih.gov/geo/query/acc.cgi?acc=GSE167924); Guo et al. 2021(GSE166422, https://www.ncbi.nlm.nih.gov/geo/query/acc.cgi?acc=GSE166422); Osnato et al. 2021(E-MTAB-10018, https://www.ebi.ac.uk/arrayexpress/experiments/E-MTAB-10018)), three studies using primed human embryonic cells giving rise to TE-like cells (one generated in house(GSE254641, https://www.ncbi.nlm.nih.gov/geo/query/acc.cgi?acc=GSE254641) ,Soncin et al. 2022(GSE182791, https://www.ncbi.nlm.nih.gov/geo/query/acc.cgi?acc=GSE182791); Ohgushi et al. 2022(GSE196365, https://www.ncbi.nlm.nih.gov/geo/query/acc.cgi?acc=GSE196365)), two studies analysing 8-cell-like cells(Mazid et al. 2022(CNP0001454, https://db.cngb.org/search/project/CNP0001454/); Yoshihara et al. 2022(E-MTAB-10581, https://www.ebi.ac.uk/arrayexpress/experiments/E-MTAB-10581)), one study with a post-implantation amniotic sac embryoid (PASE)-model(Zheng et al. 2019(GSE134571, https://www.ncbi.nlm.nih.gov/geo/query/acc.cgi?acc=GSE134571)), and one study from human trophoblast organoids(Shannon et al. 2024(GSE216244, https://www.ncbi.nlm.nih.gov/geo/query/acc.cgi?acc=GSE216244)). Furthermore, we included two embryonic datasets from Callithrix jacchus (marmoset)(Bergmann et al. 2022(E-MTAB-9367, https://www.ebi.ac.uk/biostudies/arrayexpress/studies/E-MTAB-9367); Boroviak et al. 2018(E-MTAB-7078, https://www.ebi.ac.uk/arrayexpress/experiments/E-MTAB-7078)) and three embryonic datasets from Macaca fascicularis (Crab-eating macaque)(Nakamura et al. 2016(GSE74767, https://www.ncbi.nlm.nih.gov/geo/query/acc.cgi?acc=GSE74767); Ma et al. 2019(GSE130114, https://www.ncbi.nlm.nih.gov/geo/query/acc.cgi?acc=GSE130114); Yang et al. 2021(GSE148683, https://www.ncbi.nlm.nih.gov/geo/query/acc.cgi?acc=GSE148683)).

WikiPathway annotations and gene sets for GSEA analysis were downloaded from the Molecular Signatures Database (https://www.gsea-msigdb.org/gsea/msigdb)
The processed dataset with predicted annotations, projected UMAP, and sorted cell counts, can be retrieved from https://petropoulos-lanner-labs.clintec.ki.se/dataset.download.html.

Early Embryogenesis Prediction Tool can be browsed at http://petropoulos-lanner-labs.clintec.ki.se

Human research participants

Policy information about studies involving human research participants and Sex and Gender in Research.

Reporting on sex and gender	NA
Population characteristics	NA
Recruitment	NA
Ethics oversight	NA

Note that full information on the approval of the study protocol must also be provided in the manuscript.

Field-specific reporting

Please select the one below that is the best fit for your research. If you are not sure, read the appropriate sections before making your selection.

☒ Life sciences ☐ Behavioural & social sciences ☐ Ecological, evolutionary & environmental sciences

For a reference copy of the document with all sections, see nature.com/documents/nr-reporting-summary-flat.pdf

Life sciences study design

All studies must disclose on these points even when the disclosure is negative.

Sample size	We included 42 datasets because they're relevent with this study.
Data exclusions	No data were excluded from the analyses, except that cells with unknown annotation were excluded from visualization in Fig. 1a and Extended Data Fig. 3a.
Replication	The biological replication is represented by the samples included in the datasets.
Randomization	Based on prediciton performance, downsampling sample size in Fig.2a were determined as 200. No data were excluded from the analyses. The samples were not randomized unless specified in Extended Data Fig. 3a, d and Extended Data Fig. 8a, b and d. Cells from Yang et al., 2021 were downsampled to 2000 in Extended Data Fig. 3a. Cells more than 200 cells were downsampled to 200 in Extended Data Fig. 3d and Extended Data Fig. 8a, b and d.
Blinding	Datasets were processed without taking group allocation into account.

Reporting for specific materials, systems and methods

We require information from authors about some types of materials, experimental systems and methods used in many studies. Here, indicate whether each material, system or method listed is relevant to your study. If you are not sure if a list item applies to your research, read the appropriate section before selecting a response.

Materials & experimental systems

n/a	Involved in the study	
☒	☐	Antibodies
☐	☒	Eukaryotic cell lines
☒	☐	Palaeontology and archaeology
☒	☐	Animals and other organisms
☒	☐	Clinical data
☒	☐	Dual use research of concern

Methods

n/a	Involved in the study	
☒	☐	ChIP-seq
☒	☐	Flow cytometry
☒	☐	MRI-based neuroimaging

Eukaryotic cell lines

Policy information about cell lines and Sex and Gender in Research

Cell line source(s)	Human embryonic stem cell line hs975 (KIe032-A in hPSC-reg database) was derived at Karolinska Institutet, described in Rodin S et al. Clonal culturing of human embryonic stem cells on laminin-521/E-cadherin matrix in defined and xeno-free environment. Nature communications. 2014;5:3195.
Authentication	Express pluripotency markers POU5F1 and NANOG. Trilineage differentiation has been performed.
Mycoplasma contamination	The cell line is regularly tested for mycoplasma and is indeed negative.
Commonly misidentified lines (See ICLAC register)	Not applicable
